# Diabetes health coach in individuals with type 2 diabetes: A systematic review and meta analysis of quadruple aim outcomes

**DOI:** 10.3389/fendo.2022.1069401

**Published:** 2022-12-16

**Authors:** Megan Racey, Milos Jovkovic, Paige Alliston, Muhammad Usman Ali, Diana Sherifali

**Affiliations:** ^1^ McMaster Evidence Review and Synthesis Team; and School of Nursing, Faculty of Health Sciences, McMaster University, Hamilton, Ontario, ON, Canada; ^2^ McMaster Evidence Review and Synthesis Team; and Department of Clinical Epidemiology & Biostatistics, Faculty of Health Sciences, McMaster University, Hamilton, Ontario, ON, Canada

**Keywords:** health coaching, systematic reveiw, meta analysis, quadruple aim, type 2 diabetes

## Abstract

**Background:**

As diabetes self-management necessitates life-long learning, behaviour change, support, and monitoring, health coaching is a promising intervention to assist individuals in more than just meeting glycemic goals and glycated hemoglobin (A1C) targets. Currently, studies of health coaching for type 2 diabetes (T2DM) are limited due to their emphasis on glycemic control. The goal of this systematic review and meta-analysis is to determine the effects of health coaching on adults with T2DM based on quadruple aim outcomes and to assess the implementation of these interventions.

**Methods:**

We searched 6 databases for randomized controlled trials of health coaching interventions delivered by a health professional for adults with T2DM. Reviewers screened citations, extracted data, and assessed risk of bias and certainty of evidence (GRADE). We assessed statistical and methodological heterogeneity and performed a meta−analysis of studies.

**Results:**

Nine studies were included in this review. Our meta-analysis showed a significant reduction of A1C [0.24 (95% CI, -0.38 to -0.09)] after exposure to diabetes health coaching, and small to trivial significant benefits for BMI, waist circumference, body weight, and depression/distress immediately post intervention based on moderate certainty of evidence. However, long term benefit of these clinical outcomes were not maintained at follow-up timepoints. There was a small significant benefit for systolic blood pressure which was maintained after the completion of health coaching exposure at follow-up, but there was no statistically significant benefit in other secondary outcomes such as diastolic blood pressure and lipid profile measures (e.g. triglycerides). Very few studies reported on other quadruple aim measures such as patient-reported outcomes, cost of care, and healthcare provider experience.

**Conclusions:**

Our systematic review and meta-analysis shows that health coaching interventions can have short term impact beyond glucose control on cardiometabolic and mental health outcomes. Future studies should try to examine quadruple aim outcomes to better assess the benefit and impact of these interventions at longer time points and following termination of the coaching program.

**Systematic Review Registration:**

https://www.crd.york.ac.uk/prospero, identifier (CRD42022347478).

## 1 Introduction

Diabetes is increasingly a major health issue worldwide (1), with 1 in 10 adults living with diabetes in 2021. The rise in global rates of diabetes prevalence has led to challenges in managing diabetes at the health systems and societal levels, costing 966 billion USD in health care expenditures (2). The day-to-day management of diabetes is centred around the individual and ideally supported by a multi-disciplinary team to facilitate a patient’s ability to manage one’s own diabetes care through ongoing self-management education and support (3). Self-management education and support are most effective when tailored according to: the individual’s ability for learning and readiness for change; the context of one’s cultural beliefs, health beliefs and preferences; socioeconomic barriers and other health challenges (3).

In recent years, health coaching has emerged as an effective intervention to support diabetes self-management. According to Wolever et al., health coaching may be described as: a) patient centred; b) includes patient determined goals; c) incorporates self-discovery and active learning processes; d) encourages accountability for behavioural goals; e) provides some education alongside coaching; f) a health professional who is trained in behaviour change, communication, and motivational interviewing skills (4). Health coaching may also be timely and relevant health related education, behaviour change promotion, and psychosocial support to enhance the well-being of individuals and facilitate the achievement of their health-related goals (5, 6).

Although previously marred by small pilot studies, underpowered trials, and high attrition, a growing body of quality evidence for type 2 diabetes (T2DM) suggests that individuals achieve better health outcomes with health coaching than traditional education and support programs (4, 7–9). A scan of the literature identified a few systematic reviews related to health coaching (9, 10). The first review in 2003 synthesized the effect of health coaching components; it reviewed 25 individual health coaching studies for individuals with chronic illnesses and found that while education and behaviour change are important, they are not sufficient (10). Therefore the need for coach interactions that move a patient to a stage of action were evident, as was the need to consider the emotional state of the patient (10). The second review, completed in 2010, examined the evidence for health coaching on lifestyle behaviours (9). The review included relevant studies published between 1998 and 2008, of which 15 studies included, and only 7 were randomized controlled trials (RCTs). Although the review was also not specific to diabetes health coaching, the review did note that there were significant improvements in lifestyle behaviours (diet, physical activity, weight management), as well as medication adherence. Methodological limitations were identified in the review for the 7 trials, such as small sample sizes and incomplete follow-up (9). More recently, Sherifali et al., completed a review of 8 diabetes health coaching trials and determined that coaching interventions led to an overall reduction of glycated hemoglobin (A1C) by 0.32 (95% CI, -0.50 to -0.15) (11). Exposure to diabetes health coaching for more than 6 months led to a 0.57% reduction in A1C levels (95% CI, -0.76 to -0.38), compared to exposure to a diabetes health coach for ≤6 months (−0.23%; 95% CI, -0.37 to -0.09) (11). Finally, in 2018, Pirbaglou and colleagues reviewed the literature to consider the impact of diabetes health coaching on A1C, as well as on quality of life and self-efficacy (12). Health coaching interventions were also successful in reducing A1C levels at all time points, with the largest magnitude of reduction between 4 to 9 months, but found inconsistent benefits on psychosocial findings (12).

As diabetes self-management necessitates life-long learning, behaviour change, support, and monitoring, health coaching is a promising intervention to assist individuals in more than just meeting glycemic goals and A1C targets. At present, despite the growing body of evidence, studies of health coaching for T2DM are limited due to their emphasis on glycemic control. Therefore, we will explore the literature to determine the impact of diabetes health coaching on patient-reported outcomes, clinical outcomes, provider satisfaction, and cost-effectiveness, specifically the quadruple aim goals (13). As an adaptation of a 2015 systematic review and based on the evolution of health coaching (11), the goal of this systematic review and meta-analysis is to determine the effects of health coaching on adults with type 2 diabetes based on quadruple aim outcomes and subsequently, to assess the implementation of these interventions, including describing the diabetes health coaching intervention and in what context.

## 2 Methods

This systematic review and meta-analysis followed the Preferred Reporting Items for Systematic Reviews and Meta-analyses (PRISMA) guidelines (14) from a registered protocol (PROSPERO-CRD42022347478). Our methods followed the Cochrane Handbook for Systematic Reviews of Interventions Version 6, 2019 (15).

### 2.1 Search strategy

The search terms, databases, and strategy were developed in consultation with a research librarian at McMaster University and informed by a previous systematic review (11) (**Supplemental Material 1**). We searched MEDLINE, Embase/Emcare, Cumulative Index of Nursing and Allied Health Literature (CINAHL), PsycINFO, Cochrane Database of Systematic Reviews (CDSR) and Cochrane Central Register of Controlled Trials (CENTRAL) from inception to December 2021. We manually searched reference lists of relevant reviews and included studies for citations that were not captured in our search. Results from the search were deduplicated, and citations were uploaded to a secure internet-based platform for screening (DistillerSR, Evidence Partners Inc., Ottawa, Canada).

### 2.2 Study selection and eligibility

To be included, studies had to be written in English, been published in a peer-reviewed journal, and meet the following criteria: 1) be a RCT (randomized at the patient-level); 2) report data on adults ≥18 years of age with T2DM; 3) be a health coaching intervention (beyond one-dimensional education programs) that was delivered, led, and/or implemented by a regulated healthcare professional, one who would routinely see patients with diabetes for care or management in a healthcare setting such as a clinician, nurse, or diabetes educator in primary care, community care, or hospital-based programs; and 4) include a control group which was defined as treatment as usual, standard care, or minimal contact that did not contain intervention components. There were no criteria for diagnosis of T2DM; however, studies with general adult populations or mixed populations but which have subgroup analysis for participants with T2DM were also considered. Without subgroup analysis, a mixed population must have at least 80% of participants with our targeted condition (T2DM) to be included in our review. Outcomes were not used for inclusion or exclusion of the studies. Studies were excluded if: 1) they reported data on participants younger than 18 years of age, who did not have type 2 diabetes or who were pregnant; 2) health coaching was not the primary intervention; and 3) they were not RCTs, used a quasi-randomization methodology, including cluster randomization, or were pilot or feasibility trials.

### 2.3 Data extraction and quality assessment

A team of researchers conducted the screening and data extraction (MR, MJ, PA, DS). A minimum of 2 reviewers were required to independently and in duplicate screen titles and abstracts of all potentially eligible studies. Articles marked for inclusion by either team member went on to full-text screening which was completed independently and in duplicate by 2 team members and required consensus for inclusion or exclusion. We developed, piloted, and deployed standardized forms for data extraction. For each study, one team member extracted study characteristics, risk of bias (RoB) assessment (using the Cochrane Collaboration RoB tool (16) for RCTs), template for intervention description and replication (TIDieR) checklist and guide (17), and outcome data using electronic forms housed in a web-based systematic review software program. Two team members independently verified all extracted data and disagreements were resolved through discussion and/or third party consultation. All outcomes as they relate to the Quadruple Aim framework were considered. This framework was developed to optimize health system performance and includes improved patient experience (patient-reported outcomes), better population health (clinical population health outcomes), lower costs (cost of care outcomes) and improved clinical experience (healthcare provider experience) (13). In cases where studies had multiple measures for the same outcome, we extracted the primary or direct measures before using secondary outcomes or subgroup analysis data. All extraction was independently verified by the statistician (MA). Conflicts were resolved by the lead researcher of this review (MR).

We independently evaluated the certainty of the body of evidence using the Grading of Recommendation, Assessment, Development and Evaluations (GRADE) method (18) with GRADEpro software (19). GRADE rates the certainty of a body of evidence as high, moderate, low, or very low and ratings are based on an assessment of 5 conditions: 1) methodological quality, 2) consistency across effect estimates/statistical heterogeneity, 3) directness of the body of evidence to the populations, interventions, comparators and/or outcomes of interest, 4) precision of results, and 5) indications of reporting bias.

### 2.4 Statistical analysis

All data analyses were planned a priori. A meta-analysis was used to combine the results across studies by outcome using the published data from included studies. For continuous outcomes, we used change from baseline to immediate post-treatment (means, standard deviations) and the longest follow-up data (means, standard deviations). We used a random effects multi-level meta-analytic approach to account for dependency between effect sizes (i.e., the correlation between effect sizes due to multiple measures or sub-measures of the same outcome within a study or comparison of multiple interventions to a single control group). In such cases, multiple measures and comparisons from the same study were nested within studies first and variance in observed effect sizes was decomposed into sampling variance, within study variance and between-study variance to account for intra-cluster (or intraclass) correlation in the true effects true effects (20, 21). For the pooling of patient-reported outcomes such as quality of life, depression and distress, the direction of effect was adjusted to ensure consistency of desirable outcome responses. The summary measures of effect were generated in the form of standardized mean differences (SMD) (22). The SMD is interpreted based on its magnitude according to Cohen d recommended thresholds (~0.2 = small effect, ~0.5 = medium effect, ~0.8 = large effect) (23). For studies where measure of variance was reported as confidence intervals, standard error, or p-values, we used Cochrane recommended methods to convert this data to standard deviation (24). The statistical heterogeneity I^2^ statistic was also estimated in the context of multi-level meta-analytical approach i.e., within-cluster heterogeneity (multiple comparisons from same study) and between-cluster heterogeneity (effect sizes across studies). Overall I^2^ for each summary effect size was estimated to represent the heterogeneity not attributable to sample error and is the sum of within-cluster and between-cluster heterogeneity. The Cochran’s Q (α=0.05) was employed to detect statistical heterogeneity and I² statistic to quantify the magnitude of statistical heterogeneity between studies where I² >50% represents moderate and I² >75% represents substantial heterogeneity across studies. The primary subgrouping in each analysis was based on length of follow-up i.e. immediate post-treatment and long-term follow up. All analyses were performed using R software (metafor (25) and dmetar (26) packages).

## 3 Results

**Figure 1:**
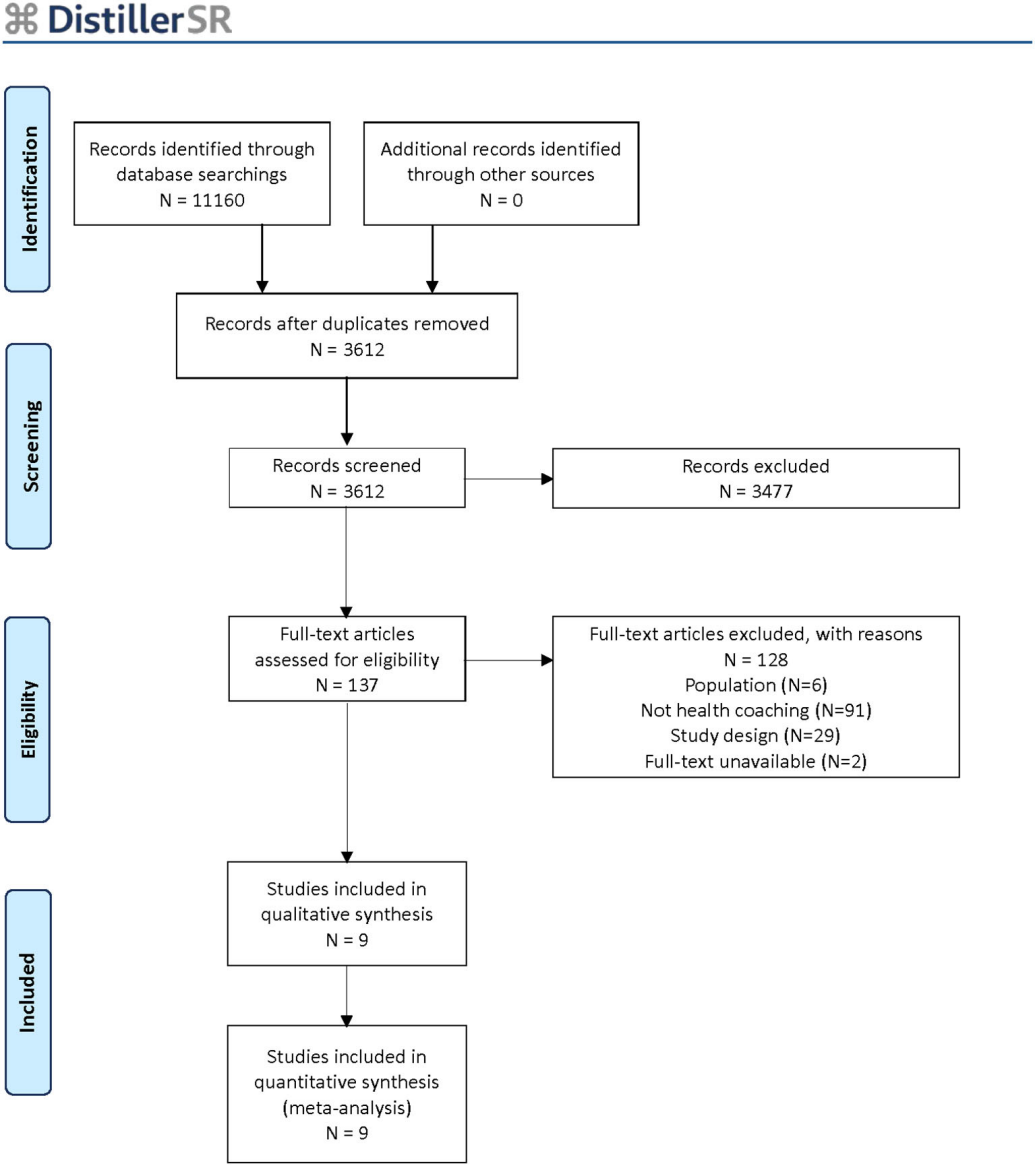
PRISMA Flow chart.

Our search strategy yielded 3,612 citations after duplicates were removed. We assessed 137 full-text articles for eligibility and included 9 RCTs in this review (**Figure 1**) (27–35). The studies were published from 2014 to 2021. We searched databases from inception and considered all studies based on the above inclusion/exclusion criteria as these criteria were updated from our similar, previous review (11) and warranted thorough screening of the literature. However, we excluded studies that were published before 2015 (one-year overlap with our previous review) which met our inclusion/exclusion criteria as they were already described in our previous review as included studies. Based on the updated inclusion/exclusion criteria of this review, some studies published before 2015 were included in this review as they were excluded in the previous review based mostly on the inclusion requirement of A1C as a reported outcome and this was no longer an exclusion criteria of this review. Likewise, some studies from our 2015 review which were described as pilot of feasibility studies were excluded in this update due to the increased rigor of our study design criteria. The PICO of this review was updated from the 2015 review to reflect the evolution of the health coaching literature and topic area.

A total sample of 2,498 adults with T2DM were included in this review with a mean age ranging from 51.0 to 66.6 years and percentage of women in the studies ranging from 10% to 78%. The mean A1C at baseline ranged from 5.5% to 9.9%. Studies were conducted across the globe in North America, Europe, and Australia, and intervention duration was between 3 months to 3 years, with most being 6 or 12 months in duration (n=7). A total of 5 studies measured outcomes beyond immediate post-treatment and 4 of these studies conducted follow-up measurements 6 months after intervention completion (29–32) while 1 study completed measurements 12 months post-intervention (28). Adverse events were only reported by 2 studies (27, 34). Balducci et al., 2019 reported any elective surgeries and medical conditions that occurred outside of the intervention and hypoglycemic episodes, arrythmias, and musculoskeletal injuries or discomfort that occurred during intervention visits or sessions (27). Sherifali et al., 2021 reported on hospitalizations (for any reason), emergency department visits, and hypo- and hyper-glycemic episodes requiring hospitalizations (34). There were no statistically significant differences in proportion of participants with adverse events between the 2 groups. Study characteristics are shown in **Table 1** and more fulsome details can be found in **Supplemental Material 2**.

**Table 1:** Characteristics of Included Studies.

Study	Balducci (2019) Italy (27)
Objective	To investigate whether a behavioral intervention strategy can produce a sustained increase in physical activity and reduction in sedentary time among individuals with type 2 diabetes (T2D).
Methods	Design: Randomized clinical superiority trial Inclusion criteria: 1) T2D for at least 1 year; 2) age 40 to 80 years; 3) body mass index of 27 to 40; 4) physical inactivity; 5) sedentary lifestyle for at least 6 months; 6) ability to walk 1.6 km without assistance; 7) and clearance by a cardiologist Exclusion criteria: not stated
Participants	Sample: N= 300; Intervention: n= 150; Control: n= 150 Follow up n: Intervention: n= 133; Control: n= 134 Mean age (SD): Overall: not stated; Intervention: 61.0 (9.7); Control: 62.3 (10.1) Gender (male): Intervention: n= 91 (60.7%); Control: n= 93 (62.0%) Race/ethnicity: not stated Mean BMI (SD): Intervention: n= 30.0 (4.9); Control: n= 30.1 (5.3) Baseline A1C % (SD): Intervention: n= 7.4 (1.6); Control: n= 7.3 (1.4)
Intervention	Intervention duration: 3 years Description of intervention: 1 individual theoretical counseling session, conducted by a diabetologist, and 8 biweekly individual theoretical and practical counseling sessions, conducted by a certified exercise specialist, per year. Description of control group: General physician recommendations for increasing daily physical activity and decreasing sedentary time. Length of follow up: NA
Quadruple Aims Outcomes	Clinical population health
**Study**	**Cummings** (2019) **USA** (33)
Objective	To evaluate the effect of cognitive behavioral therapy (CBT) plus lifestyle counseling in primary care on hemoglobin A1c (HbA1c) in rural adult patients with T2D and comorbid depressive or regimen-related distress (RRD) symptoms.
Methods	Design: Randomized controlled trial Inclusion criteria: 1) adult patients (18–75 years) with a medical record–established history of T2D with an HbA1c at screening >7.0% (53 mmol/mol) and with a positive screen for symptoms of distress using the Diabetes Distress Scale 2 (DDS-2) item screener and/or a positive screen for symptoms of depression on the Patient Health Questionnaire 2 (PHQ-2) item screener Exclusion criteria: 1) medical record–established diagnosis of advanced disease or the presence of alcoholism, cognitive impairment, or major psychiatric illness that would preclude active participation
Participants	Sample: N= 139; Intervention: n= 67; Control: n= 72 Follow up n: Intervention: n= 58; Control: n= 62 Mean age (SD): Overall: 52.6 (9.6); Intervention: 51.0 (9.0); Control: 53.0 (9.0) Gender (male): Intervention: n= 14 (21%); Control: n= 17 (24%) Race/ethnicity (%): African American: I: 77; C: 67 Mean BMI (SD): not stated Baseline A1C % (SD): Intervention: n= 9.88 (2.1); Control: n= 9.35 (1.7)
Intervention	Intervention duration: 12 months Description of intervention: The CBT subgroup intervention focused on the reduction of depressive and/or RRD symptoms through modification of negative thoughts and problematic behaviors as well as improvement of diabetes self-management strategies. Sessions were delivered by a clinical health psychologist as well as a doctoral student in clinical health psychology. CBT intervention components were guided by two evidence-based treatment manuals for behavioral activation. Description of control group: Standard care Length of follow up: NA
Quadruple Aims Outcomes	Patient-reported/experience; Clinical population health
**Study**	**Jutterström** (2016) **Sweden** (31)
Objective	To evaluate the effect of a patient-centered self-management support, in T2D with regard to metabolic changes
Methods	Design: Randomized controlled trial Inclusion criteria: 1) diagnosed with T2D within three years; 2) aged 40–80 years; 3) Swedish speaking; 4) and no diagnosed cognitive impairment or other severe illnesses; 5) had not received patient education other than information given to newly diagnosed T2D patients Exclusion criteria: not stated
Participants	Sample: N= 195; Intervention: n= 70, 35; Control: n= 36; External Control (EC): n=54 Follow up n: Intervention: n= 59, 33; Control: n= 32; EC: n= 47 Mean age (SD): Overall: 64.5 (9.58); Intervention: 64.0 (8.72), 64.9 (11.10); Control: 62.6 (10.61); EC: 66.2 (8.75) Gender (male): Intervention: n= 43 (68.3%), 21 (61.8%); Control: n= 18 (52.9%); EC: n= 32 (62.7%) Race/ethnicity: not stated Mean BMI (SD): Intervention: n= 30.22 (5.22), 31.76 (5.73); Control: n= 30.56 (5.81); EC: n= 29.62 (5.27) Baseline A1C % (SD): Intervention: n= 6.0 (0.93), 5.8 (0.87); Control: n= 5.8 (0.77); EC: n= 5.5 (0.84)
Intervention	Intervention duration: 6 months Description of intervention: Participants in the group intervention (GI) and individual intervention (II) groups were invited to six sessions of 45–90 min each. In the GI group, the patients reflected aspects of living with type 2 diabetes together and the diabetes specialist nurses (DSNs) acted as a moderator. The II participants met the local diabetes nurse one-on-one. Description of control group: Standard care Length of follow up: 12 months#
Quadruple Aims Outcomes	Clinical population health
**Study**	**Karhula** (2015) **Finland** (35)
Objective	To study whether a structured mobile phone-based health coaching program, which was supported by a remote monitoring system, could be used to improve the health-related quality of life (HRQL) and/or the clinical measures of T2D and heart disease patients.
Methods	Design: Randomized controlled trial Inclusion criteria: 1) diagnosis of T2D, glycosylated hemoglobin (HbA1c) level, which needed to be above 6.5% within 1 year prior to the screening; 2) diagnosed with diabetes at least 3 months earlier; 3) 18 years of age or older; 4) ability to fill in questionnaires in Finnish; 5) ability to use the RPM system and the devices provided; 6) having adequate cognitive capacities to participate, being able to walk Exclusion criteria: not stated
Participants	Sample: N= 287; Intervention: n= 208; Control: n= 79 Follow up n: Intervention: n= 162; Control: n= 63 Mean age (SD): Overall: not stated; Intervention: 66.6 (8.2); Control: 65.5 (9.6) Gender (male): Intervention: n= 99 (55%); Control: n= 40 (57%) Race/ethnicity: not stated Mean BMI (SD): Intervention: n= 31.1 (5.4); Control: n= 30.9 (5.7) Median Baseline A1C %: Intervention: n= 7.25; Control: n= 7.20
Intervention	Intervention duration: 12 months Description of intervention: Health coaching over mobile phones and self-monitoring of health parameters with the help of a remote patient monitoring (RPM) system. Description of control group: Standard care Length of follow up: NA
Quadruple Aims Outcomes	Patient-reported/experience; Clinical population health
**Study**	**Naik** (2019) **USA** (30)
Objective	To evaluate the effectiveness of proactive population screening plus telephone delivery of a collaborative goal-setting intervention among high-risk patients with uncontrolled diabetes and depression.
Methods	Design: Randomized clinical trial Inclusion criteria: 1) Veterans with uncontrolled diabetes (defined by International Classification of Diseases and HbA1c of > or =7.5% for 1 year before the study) who lived at least 20 miles from the main Veterans Health Administration hospital in Houston, Texas, or who received primary care services within a MEDVAMC satellite community-based clinic across Southeast Texas Exclusion criteria: 1) severe cognitive impairment or mental health condition; 2) hearing or visual impairment; 3) active suicidal ideation; 4) presence of significant hypoglycemic events; 5) substance abuse
Participants	Sample: N= 225; Intervention: n= 136; Control: n= 89 Follow up n: Intervention: n= 90; Control: n= 68 Mean age (SD): Overall: 61.9 (8.3); Intervention: not stated; Control: not stated Gender (male): Intervention: n= 121 (89%); Control: n= 81 (91%) Race/ethnicity n (%): White - I: 73 (53.7); C: 51 (57.3); non-Hispanic black - I: 41 (30.1); C: 16 (18.0); Hispanic - I: 12 (8.8); C: 11 (12.4); Other - I: 10 (7.4); C: 11 (12.4) Mean BMI (SD): not stated Baseline A1C % (SD): Intervention: n= 9.2 (1.4); Control: n= 9.3 (1.5)
Intervention	Intervention duration: 6 months Description of intervention: Nine sessions across 6 months in which coaches focused on goal setting, discrete skill modules (increasing pleasant activities, using thoughts to improve wellness, diet, physical activity, medication management, and relaxation) and maintenance skills customized to meet their diabetes and depression goals. Description of control group: Usual care Length of follow up: 12 months#
Quadruple Aims Outcomes	Patient-reported/experience; Clinical population health; Cost of care/system-level
**Study**	**Odnoletkova** (2016) **Belgium** (28)
Objective	To investigate the effect of the COACH programme on HbA1c and other modifiable diabetes risk factors in people with T2D in a primary care setting.
Methods	Design: Randomized controlled trial Inclusion criteria: 1) adults aged 18–75 years; 2) diagnosis of T2D; 3) received glycose-lowering oral and/or injectable therapy Exclusion criteria: 1) corticoid therapy and/or a debilitating coexisting medical condition, such as dialysis, mental illness or cancer; 2) residence in long-term care facilities; 3) pregnancy; 4) insufficient proficiency in Dutch
Participants	Sample: N= 574; Intervention: n= 287; Control: n= 287 Follow up n: Intervention: n= 240; Control: n= 246 Mean age (SD): Overall: not stated; Intervention: 63.8 (8.7); Control: 62.4 (8.9) Gender (male): Intervention: n= 173 (60%); Control: n= 180 (63%) Race/ethnicity: not stated Mean BMI (SD): Intervention: n= 30.2 (4.9); Control: n= 30.6 (5.2) Baseline A1C % (SD): Intervention: n= 7.0 (1.0); Control: n= 7.9 (0.9)
Intervention	Intervention duration: 6 months Description of intervention: The underlying ‘COACH model’ is a continuous quality improvement cycle, which includes bridging the knowledge gap, assertiveness training, setting an action plan and (re)assessment. The COACH programme consisted of five telephone sessions of a mean (range) duration of 30 (10–45) min, delivered at a mean (range) interval of 5 (3–8) weeks by a certified diabetes nurse educator. Description of control group: Usual care Length of follow up: 18 months#
Quadruple Aims Outcomes	Patient-reported/experience; Clinical population health; Healthcare provider experience; Cost of care/system-level
**Study**	**Sherifali** (2021) **Canada** (34)
Objective	To evaluate the effect of a 12-month telephone diabetes health coaching (DHC) intervention on glycemic control in persons living with T2D.
Methods	Design: Community-based randomized controlled trial Inclusion criteria: 1) ≥18 years of age; 2) T2D diagnosis; 3) A1C level >7.5% within 6 months before randomization; 4) ability to read and write in English; 5) telephone access Exclusion criteria: 1) pregnancy; 2) debilitating coexisting conditions (i.e., mental illness, impaired cognition); 3) underlying medical conditions that could provide misleading A1C levels
Participants	Sample: N= 365; Intervention: n= 188; Control: n= 177 Follow up n: Intervention: n= 186; Control: n= 171 Mean age (SD): Overall: not stated; Intervention: 56.82 (11.69); Control: 59.05 (11.79) Gender (male): Intervention: n= 99 (52.66%); Control: n= 83 (46.89%) Race/ethnicity n (%): Caucasian: I: 150 (79.79); C: 144 (81.36) Mean BMI (SD): Intervention: n= 34.71 (7.80); Control: n= 35.36 (8.35) Baseline A1C % (SD): Intervention: n= 9.10 (1.65); Control: n= 8.86 (1.50)
Intervention	Intervention duration: 12 months Description of intervention: Diabetes health coaching comprised of care that included: 1) case management and monitoring; 2) diabetes self-management education and support; 3) behaviour modification, goal setting and reinforcement; and 4) general psychosocial support. Description of control group: Usual diabetes education (individual or group) provided by nurses and/or dietitians, typically every 3 to 6 months, along with community resources and a study provided accelerometer. Length of follow up: NA
Quadruple Aims Outcomes	Patient-reported/experience; Clinical population health
**Study**	**Varney** (2014) **Australia** (32)
Objective	To measure the effect of a 6-month telephone coaching intervention on glycaemic control, risk factor status and adherence to diabetes management practices at the intervention’s conclusion (6 months) and at 12 months.
Methods	Design: Randomized controlled trial Inclusion criteria: 1) adults with T2D and HbA1C >7% Exclusion criteria: 1) unable to provide consent; 2) non-English speaking; 3) cognitively impaired; 4) receiving palliative care; 5) severely hearing impaired or without telephone access
Participants	Sample: N= 94; Intervention: n= 47; Control: n= 47 Follow up n: Intervention: n= 35; Control: n= 36 Mean age (95% CI): Overall: not stated; Intervention: 59 (56–62); Control: 64 (61–66) Gender (male): Intervention: n= 34 (72%); Control: n= 30 (64%) Race/ethnicity n (%): Caucasian I: 46 (98); C: 37 (79); Asian/Indian I: 1 (2); C: 8 (17); Afro-Caribbean I: 0 (0); C: 2 (4) Mean BMI (95% CI): Intervention: n= 32.1 (30.3-33.9); Control: n= 30.9 (29.1-32.6) Baseline A1C % (SD): Intervention: n= 8.2 (8.0-9.7); Control: n= 8.5 (8.1-8.9)
Intervention	Intervention duration: 6 months Description of intervention: Participants were encouraged to follow a low saturated fat, high-fibre diet, with 50% of energy from carbohydrates, and were encouraged to exercise for 150 min per week. During subsequent telephone coaching sessions, progress towards treatment goals, risk factor status, adherence to self-care and monitoring requirements were reassessed. If goals were not achieved, barriers to goal attainment were identified, an action plan addressing these barriers was agreed and new goals were established. Description of control group: Received telephone calls to arrange baseline, 6- and 12-month assessment appointments. Could access usual care services, including a diabetes clinic staffed by endocrinologists, diabetes educators and dietitians. Length of follow up: 12 months#
Quadruple Aims Outcomes	Patient-reported/experience; Clinical population health
**Study**	**Young** (2020) **USA** (29)
Objective	To evaluate the effectiveness of a nurse coaching program using motivational interviewing paired with mobile health (mHealth) technology on diabetes self-efficacy and self-management for persons with T2D.
Methods	Design: Randomized controlled trial Inclusion criteria: 1) aged 18 years or above; 2) receiving care at 1 of the 3 clinics; 3) living with T2D and having HbA1c of 6.5% (48 mmol/mol) or higher; and 4) able to speak English Exclusion criteria: 1) no access to a telephone; 2) were not able to consent because of cognitive impairment, or were pregnant
Participants	Sample: N= 319; Intervention: n= 158; Control: n= 161 Follow up n: Intervention: n= 132; Control: n= 155 Mean age (SD): Overall: 59.07 (11.4); Intervention: 58.96 (11.3); Control: 59.18 (11.5) Gender (male): Intervention: n= 81 (52.6%); Control: n= 84 (52.8%) Race n (%) - Caucasian I: 96 (63.2); C: 100 (62.9), African American I: 21 (13.8); C: 18 (11.3), Asian I: 11 (7.2); C: 16 (10.1), Other I: 16 (10.5); C: 14 (8.8), More than 1 race I: 8 (5.3); C: 11 (6.9) Ethnicity n (%) - Hispanic or Latino I: 24 (17.5); C: 18 (12.9), Not Hispanic or Latino I: 113 (82.5); C: 122 (87.1) Mean BMI (SD): not stated Baseline A1C % (SD): not stated
Intervention	Intervention duration: 3 months Description of intervention: Each participant was paired with a nurse health coach who delivered 6 individual sessions using a counseling style based on the concepts of motivational interviewing. Sessions were structured to promote mutual goal setting, enhance self-efficacy in health behaviour change, and assist individuals to derive meaning from the data to reinforce choices and behaviours. Description of control group: Usual care comprised standard health care visits with providers and access to classes, resources, and services (i.e., diabetes management and weight loss education, electronic learning videos, and care coordination). Length of follow up: 9 months#
Quadruple Aims Outcomes	Patient-reported/experience

NA, not applicable.

#, follow up reported as time from baseline.

### 3.1 Diabetes coaching intervention characteristics

The objective or rationale for the health coaching interventions was either to directly affect glycemic control or to influence glycemic control and/or diabetes management through other self-care behaviours and reducing risk factors (**Table 1**). As per our inclusion criteria, all studies used healthcare professionals to deliver the health coaching intervention. For most studies (n=7), just one type of coach was used but in 2 studies (30, 33), a team of health professionals worked together for the delivery of different components of the intervention. Coaches across the studies included a certified diabetologist, nurses, psychologists, doctoral students, community health workers, pharmacists, social workers, certified diabetes nurse educators, and a dietitian (**Supplemental Material 2**).

Coaching interventions were deployed using various strategies (**Supplemental Materials 2, 3**). Telephone-only strategies were used by 6 studies (28, 30, 32–35), while telephone and face-to-face was used in 1 study (29), and 2 studies used in-person or face-to-face strategies only (27, 31). All the studies were focused on individual or one-on-one interactions and only 1 study also included group components. Sessions and interactions with the coaches ranged from weekly, to bi-weekly, to as infrequent as one session every 4 to 6 weeks. The duration of these sessions also varied from as short as 15 minutes to as long as 90 minutes; however, most seemed to average around 30 minutes. Any in-person components of the health coaching interventions took place in outpatient healthcare settings such as clinics, centres, primary care offices, and doctors offices (**Supplemental Material 3**). One study did not provide any details about the location of the intervention beyond geographical area, but it was a telephone/virtual program (34). The fact that over 60% of the included studies consisted of telephone-only interactions means that many of the intervention components and exchanges between the coaches and participants occurred from wherever the participant was at that time.

**Figure 2:**
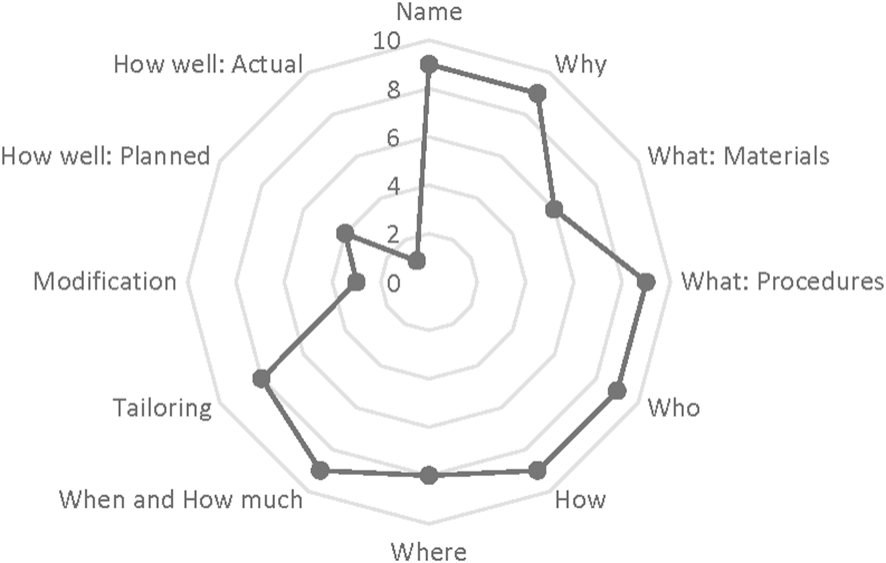
Spider chart of the total reporting of TIDieR Tool Items.

We described the diabetes health coaching intervention using the TIDieR checklist (17); reporting on the 12 domains were inconsistent with limited reporting on implementation fidelity (planned vs. actual) (**Figure 2**). Almost all the included studies specifically mentioned tailoring and personalization of the intervention to the participants needs (n=8), which is likely reflective of the personal nature of health coaching (**Figure 2**). While Balducci et al., did consist of individual face-to-face sessions with the coach (27), there was no explicit mention of how these sessions were tailored to the participant. Three studies mentioned the modification of the intervention from what was originally planned. Balducci et al., created a two-step scaled intensity for physical activity to support behaviour change (27). Based on feedback and study measures, Karhula et al., adjusted the length coaching phone calls to be shorter in duration (35). Young et al., had an unexpected recall of equipment that resulted in the use of a different activity tracker (29). Moreover, very few studies assessed how well the intervention was delivered (**Figure 2**). Four studies outlined plans to assess intervention fidelity, consisting of quality control measurements, listening to recordings of participant-coach interactions, and auditing of sessions, but only 1 included this as an outcome in their paper. Odnoletkova et al., conducted interviews with healthcare providers to assess intervention implementation (28). It is important to note that this component of TIDieR goes beyond attendance and adherence of the intervention by participants.

Studies were also mapped to the proposed Sherifali Diabetes Coaching Model (36). This model is comprised of four components: (i) personal case management and monitoring, emphasizing process of care issues and system navigation related to diabetes; (ii) diabetes self-management education and support, highlighting the need for knowledge, skill acquisition, and problem solving related to day-today management; (iii) behaviour modification, goal setting and reinforcement, using motivational interviewing and theories to facilitate goal setting, attainment, and behaviour change; and (iv) general psychosocial support, leveraging active listening and empathy to provide support. The studies in our review all included intervention components related to self-management and education (n=9) and almost all the studies also addressed behaviour modification (n=8). Psychosocial support was included in 66% of the studies (n=6), while only one third of the studies addressed personal case management and monitoring.

### 3.2 Risk of bias and quality of included studies

The Cochrane RoB tool showed mixed quality of study methodology: 4 studies were low risk of bias (27–30), 4 were unclear risk of bias (31–34), and 1 was high risk of bias (35) mostly due to issues regarding blinding of participants, providers, and/or outcome assessment (**Table 2**).

**Table 2:** Risk of bias.

Author, year (ref)	SEQUENCE GENERATION	ALLOCATION CONCEALMENT	BLINDING OF PATIENTS/PARTICIPANTS & PROVIDERS/PERSONNEL	BLINDING OF OUTCOME ASSESSMENT	INCOMPLETE OUTCOME DATA	SELECTIVE REPORTING	OTHER BIAS
**Balducci 2019** (27)	L	L	H	L	L	L	L
**Cummings 2019** (33)	L	L	U	U	L	L	L
**Jutterström 2016** (31)	L	L	U	U	U	L	L
**Karhula 2015** (35)	L	L	U	U	H	L	L
**Naik 2019** (30)	L	L	U	L	L	L	L
**Odnoletkova 2016** (28)	L	L	U	L	L	L	L
**Sherifali 2021** (34)	L	L	U	U	L	L	L
**Varney 2014** (32)	L	L	H	U	L	L	L
**Young 2020** (29)	L	U	L	U	L	L	L

U=unclear risk.

L=low risk.

H=high risk.

The certainty of evidence, as assessed by GRADE, ranged from very low to moderate due to concerns regarding risk of bias (studies rated as unclear risk of bias), inconsistency (direction of effect is not consistent with substantial heterogeneity observed across studies), and imprecisions (inadequate sample size and imprecise effect estimates with confidence intervals the include no effect) (**Supplemental Material 4**).

### 3.3 Benefits of treatment

We extracted and categorized outcomes based on the Quadruple Aim framework (13) and were able to meta-analyze outcomes for patient-reported and clinical population health outcomes. For the remaining outcomes, there was insufficient data and number of studies for meta-analysis, therefore, these are described narratively.

#### 3.3.1 Patient-reported outcomes

**Figure 3:**
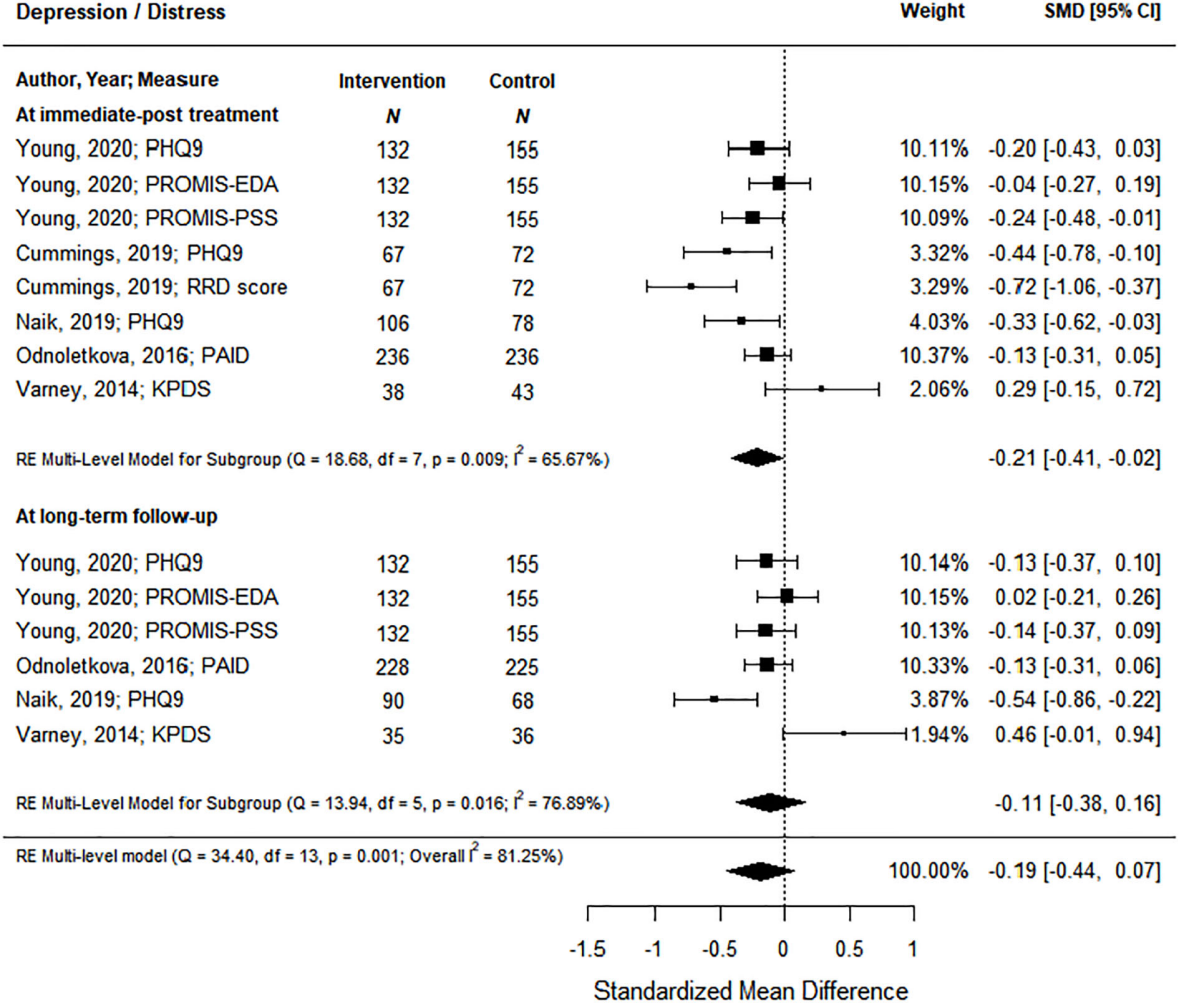
The effect of coaching interventions on depression/distress.

Patient-reported outcomes included satisfaction, diabetes empowerment, quality of life, and depression/distress. Only 1 study reported on satisfaction using the Diabetes Treatment Satisfaction Questionnaire (28) and 1 reported on empowerment with the Diabetes Empowerment Scale (29). Quality of life outcomes were reported by 4 studies post-intervention and 2 studies at long-term follow-up using a variety of standardized tools/questionnaires; however, there was no significant effects between intervention or control groups at either time point (**Supplemental Material 5**). Five studies reported the effect of the coaching intervention on depression/distress. At immediate post-treatment, health coaching interventions showed a significant decrease of small magnitude in depression/distress levels of 0.21 (95% CI, -0.41 to -0.02) based on moderate certainty of evidence (**Figure 3**; **Supplemental Material 4**). At long-term follow-up, the 4 studies with data did not show a significant effect.

#### 3.3.2 Clinical population health

**Figure 4:**
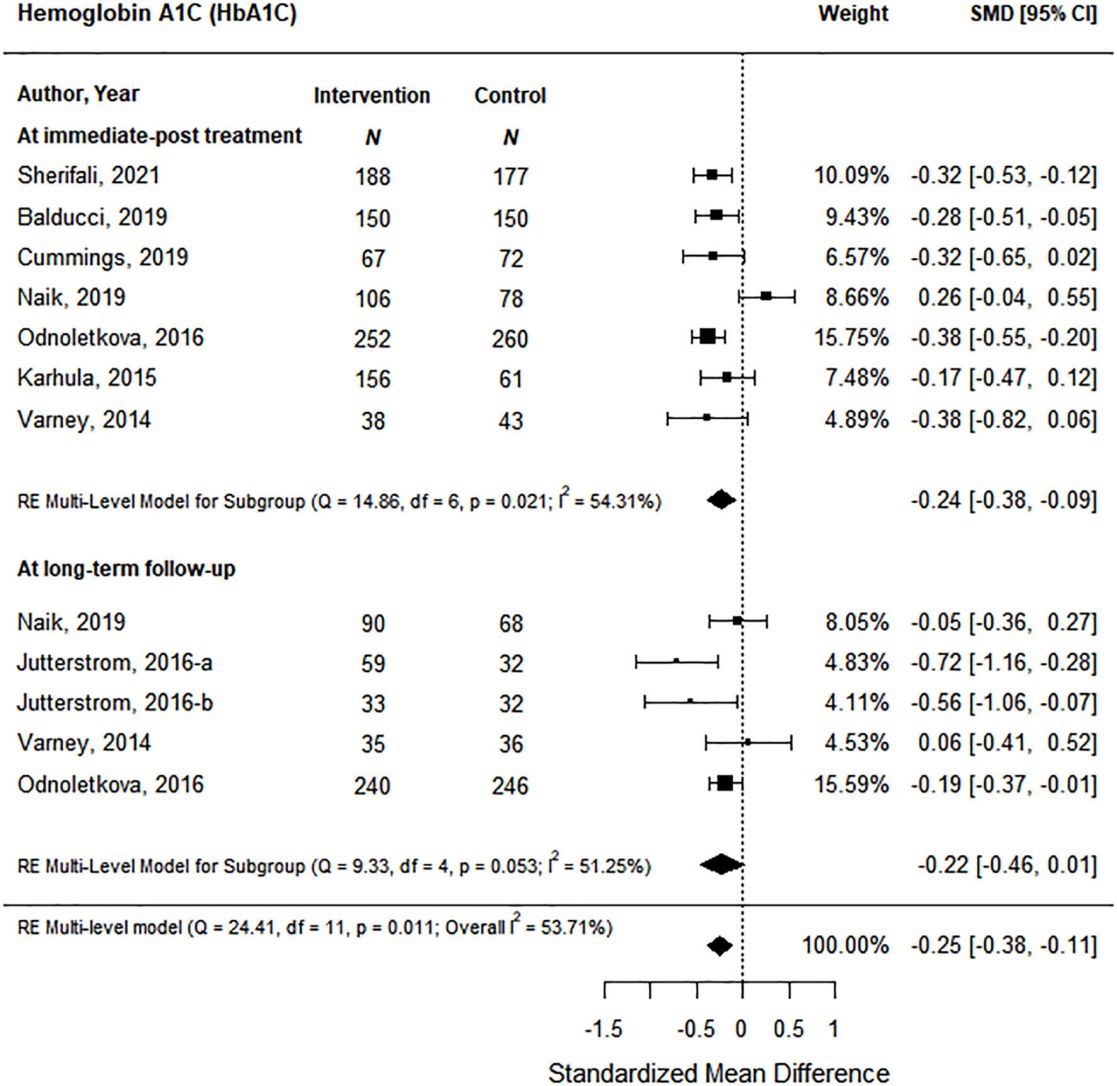
The effect of coaching interventions Hba1c levels.

Glucose control was measured by glycosylated hemoglobin (A1C) and fasting blood glucose (FBG). Eight of the nine studies reported the effect of the coaching intervention on A1C levels. At immediate post-treatment, data from 7 health coaching interventions showed a significant decrease of small magnitude in A1C levels of 0.24 (95% CI, -0.38 to -0.09) based on moderate certainty of evidence (**Figure 4**; **Supplemental Material 4**). At long-term follow-up, the 4 studies with A1C data did not show a significant effect. FBG was reported in 2 studies (27, 32). Immediately post-treatment, both studies found significant improvements in FBG; however, this effect was not maintained at long-term follow-up in Varney et al.

**Figure 5:**
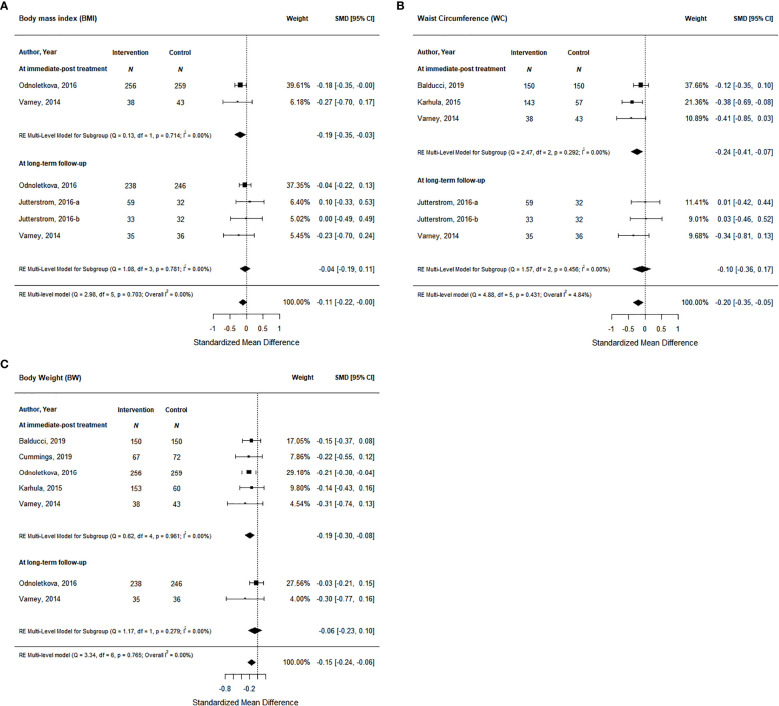
The effort of coaching interventions on anthropometric outcomes (**(A)**. BMI; **(B)** Waist circumtances; **(C)** Body Weight).

Studies also measured anthropometric outcomes including body mass index (BMI), waist circumference, and body weight. Two studies measured BMI post-intervention and 3 studies measured BMI at long-term follow-up. At immediate post-treatment, health coaching interventions showed a significant decrease of small magnitude in BMI of 0.19 (95% CI, -0.35 to -0.03) based on moderate certainty of evidence (**Figure 5A**; **Supplemental Material 4**); however, this effect was not maintained at long-term follow-up. Similarly, at immediate post-treatment, data from 3 health coaching interventions showed a significant decrease in waist circumference of small magnitude of 0.24 (95% CI -0.41 to -0.07) based on moderate certainty of evidence (**Figure 5B**; **Supplemental Material 4**); however, this effect was not maintained at long-term follow-up with data from 2 studies. Lastly, the data from 5 studies on body weight at post-intervention and 2 studies at long-term follow-up showed similar trends. At immediate post-treatment, health coaching interventions showed a significant decrease of small magnitude in body weight of 0.19 (95% CI -0.30 to -0.08) based on moderate certainty of evidence (**Figure 5C**; **Supplemental Material 4**). At long-term follow-up, the data did not show a significant effect.

**Figure 6:**
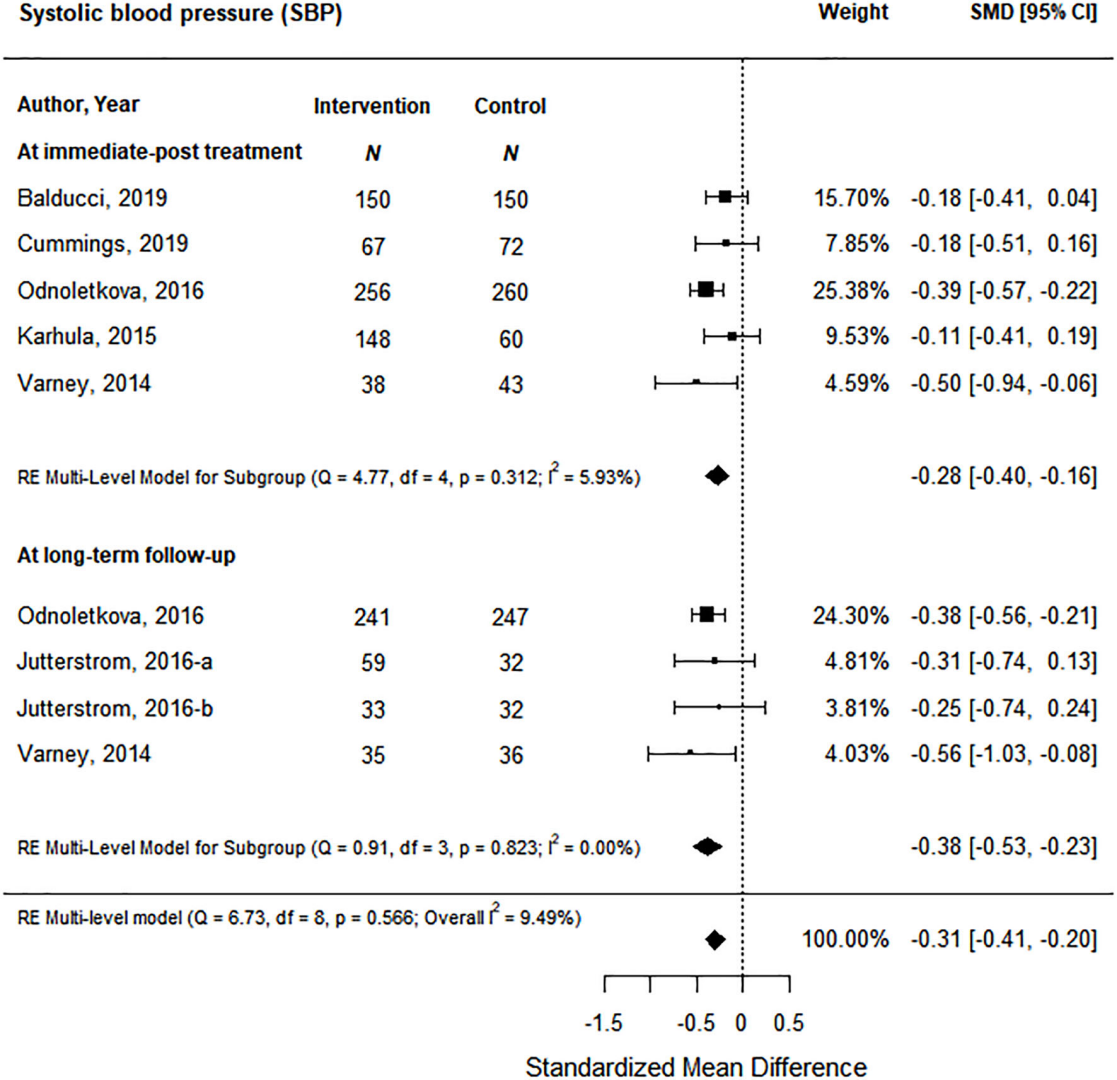
The effect of coaching interventions on systolic blood pressure.

Blood pressure was reported as both systolic and diastolic in our included studies. At immediate post-treatment, data from 5 health coaching interventions showed a significant decrease of small magnitude in systolic blood pressure of 0.28 (95% CI -0.40 to -0.16) based on moderate certainty of evidence (**Figure 6**; **Supplemental Material 4**) and this effect was maintained at long-term follow-up based on data from 3 studies with a significant decrease of small magnitude of 0.38 (95% CI -0.53 to -0.23) based on moderate certainty of evidence (**Figure 6**; **Supplemental Material 4**). Diastolic blood pressure data came from 4 studies at post-treatment and 3 studies at long-term follow-up; however, health coaching interventions did not show significant effects at either time point (**Supplemental Material 5**).

Lastly, many of our included studies measured other cardiometabolic outcomes from blood triglycerides and cholesterol levels. Data from 4 studies at immediate post-treatment and 3 studies at long-term follow-up showed no significant effects from the coaching interventions on total cholesterol, low density lipoprotein (LDL), high density lipoprotein (HDL), or triglycerides (**Supplemental Material 5**).

#### 3.3.3 Cost of care

Outcomes related to cost of care were measured in 2 studies (28, 30) as the use of health care services. Both studies measured clinic visits including mental health clinic visits, primary care/general practitioner clinic visits, and other healthcare specialist visits such as endocrinologists, cardiologists, and ophthalmologists. Naik et al., did not see any significant differences in health care use or clinic visits between treatment and control groups, but Odnoletkova did find significant differences between treatment and control groups. Those randomized to the coaching intervention sought out healthcare services and specialist visits and tests more than those in the control group. There was no data from our included studies on the cost of care such as cost of the coaching interventions, or any cost savings due to the intervention programs.

#### 3.3.4 Healthcare provider experience

Odnoletkova was the only study in our included articles to report on healthcare provider experience and this was described in a mixed-method study embedded in their clinical trial (37). Through both questionnaires and interviews, the study explored the perceptions of participants, nurses, and general practitioners (GPs) regarding the telecoaching intervention. Overall, both GPs and nurses found the coaching intervention to be sufficient and facilitated their work and noted that diabetes education is lacking in their training. According to most GPs, work still needs to be done to identify the groups of patients who would benefit from coaching programs using different methods (i.e. in person vs telephone). Healthcare providers agreed that a combination of phone and face-to-face consultations is necessary.

## 4 Discussion

We examined the literature to determine the impact of diabetes health coaching on the quadruple aim outcomes of patient-reported outcomes, clinical outcomes, provider satisfaction, and cost-effectiveness, and to describe the implementation and context of diabetes health coaching interventions. A total of 9 trials from Europe, Australia, and North America were included and the description of what comprised diabetes health coaching as an intervention was consistent across studies and previously published literature (11, 12). While 8 of the 9 studies reported on the impact of diabetes health coaching on glycemic control, there was limited evidence on patient-reported outcomes, provider satisfaction, and cost of care.

Similar to other reviews (11, 12, 38), our review of the literature found that diabetes health coaching interventions are still diverse with respect to the delivery personnel, mode of delivery, and most notably when it comes to the frequency, duration, and location of coaching interactions and sessions. The differences in the mode of delivery and length of interventions raises the need for further research into which study-level factors are most effective for all the quadruple aim outcomes. There was great variability in the individual components of the coaching interventions; however, they still aligned well to the Sherifali Coaching model (36) and definitions of health coaching more broadly (4–6). The reporting of intervention components also aligned with standard reporting guidelines such as CONSORT (39). The studies in our review provided details related to the why, what, who, how, where, and when of diabetes health coaching interventions, but components related to intervention fidelity were lacking.

Our meta-analysis showed a statistically and clinically significant reduction of A1C [0.24 (95% CI, -0.38 to -0.09)] after exposure to diabetes health coaching, and small to trivial benefits for BMI, waist circumference, body weight, and depression/distress at post treatment. However, long term benefit was not seen across all clinical outcomes, following the completion of the diabetes health coaching intervention. Although there was a small benefit noted for systolic blood pressure, which was maintained following diabetes health coaching exposure, there was no statistically significant benefit in other secondary outcomes such as diastolic blood pressure and lipid profile measures (e.g. triglycerides). It is important to note that there is heterogeneity and imprecision in the health coaching interventions and associated data from our included studies which downgrades our confidence in the generalizability of these treatment effects and lowers the overall certainty of evidence rating (GRADE) for specific outcomes. In such cases, these results and their generalizability should be interpreted with caution, warranting future high quality research with adequate sample sizes to further affirm the findings of our systematic review and meta-analysis.

The pooled treatment effect of diabetes health coaching on A1C is smaller in magnitude than previous reviews which have estimated approximately a 0.5% reduction in A1C following a six month diabetes health coaching intervention (11, 12). The decrease in pooled treatment effect size in our review may be due to the changing nature of diabetes self-management, with many diabetes clinical guidelines and standards of practice placing a greater emphasis on self-management support, particularly starting around 2015 (3). Furthermore, as the development, implementation, and evaluation of diabetes health coaching as an intervention gains greater attention, fewer pilot studies are being conducted. Pilot studies often yield larger and less precise effect sizes, whereas full-scale RCTS with powered sample sizes and larger trials are yielding more precise, real-world estimates of diabetes health coaching in a variety of contexts. However, longer duration intervention studies are required to fully assess the implementation, impact on clinical population health outcomes, cost of care, sustainability, and legacy effect of diabetes health coaching since other reviews also found a reduced effectiveness of these interventions with longer study durations beyond one year (12).

The examination of quadruple aim goals in the context of diabetes health coaching demonstrated a paucity of evidence. Specifically, clinical outcomes related to blood pressure and lipid management may require longer duration studies, beyond 6 months, to yield observed clinical benefits. Other reviews have also failed to find a significant benefit of health coaching interventions for these cardiometabolic clinical outcomes (38). Beyond these clinical outcomes, we also found there was limited evidence on patient-reported outcomes, provider satisfaction, and cost of care. The few studies that did report on these outcomes showed mixed results, and while we found a significant decrease of small magnitude in depression/distress levels of 0.21 (95% CI, -0.41 to -0.02), other reviews have not seen the same significance in their analyses (12, 38). One explanation for these findings may be the emphasis that diabetes health coaching interventions currently place on metabolic control of T2DM. The patient-reported outcomes may require more psychologically focussed programming. Based on the Sherifali Coaching model (36), we found psychosocial support was included in only 66% of the studies (n=6), while only one third of the studies addressed personal case management and monitoring.

Our review was comprehensive, having searched multiple databases and leveraged a comprehensive search strategy from a previous review. However, we did not search grey literature and we only included studies that met our predetermined inclusion criteria. We have noted that there is heterogeneity in our included studies but unfortunately, we could not perform any meta-regression analysis based on study-level factors (such as coaching intervention type, length of intervention, or population) as there were too few studies to conduct such analysis. We also applied a transparent definition of diabetes health coaching which may be excluding studies in which diabetes health coaching was delivered by a non-health care professional. However, by applying the TIDieR checklist to the descriptions of the study interventions, we are contributing to the greater understanding of what comprises diabetes health coaching.

## 5 Conclusions

Findings from this systematic review and meta-analysis showed that the diabetes health coaching literature continues to evolve, adding more evidence from larger trials of longer duration on the benefit on glucose control (such as A1C). Health coaching interventions can have short term impact beyond glucose control on cardiometabolic and mental health outcomes. As diabetes health coaching continues to be implemented as a self-management support intervention, future research should continue to explore the impact of health coaching on patient reported outcomes, other metabolic health outcomes, provider satisfaction, and cost to better assess the impact of these interventions at longer time points following the termination of the coaching program. More broadly, this systematic review provides a road map of gaps and opportunities for future research in diabetes health coaching evaluation and implementation.

## Data availability statement

The original contributions presented in the study are included in the article/**Supplementary Material**. Further inquiries can be directed to the corresponding author.

## Author contributions

All authors were involved in conception and design of the study and approved the protocol; MR, DS were responsible for overseeing the search of databases and literature. MR handled management of database and deduplication or records. MR, MJ, PA, DS were involved in the screening of citations; MR, MJ, PA were responsible for data extraction; MR, MA, DS were responsible for data verification and analysis of data. MR, MA, DS were involved in interpretation of data. All authors supported in the drafting of the manuscript which was led by MR and all authors supported in revising and formatting of the manuscript. All authors contributed to the article and approved the submitted version.
