# Supplementary material for: Diabetes health coach in individuals with type 2 diabetes: A systematic review and meta analysis of quadruple aim outcomes
Source: Front Endocrinol (Lausanne). 2022 Dec 16;13:1069401. doi: 10.3389/fendo.2022.1069401 (PMC9800616; doi:10.3389/fendo.2022.1069401)
Supplement: Supplementary file 1 [file DataSheet_1.docx]

Supplementary Material

# Search Strategy

MEDLINE

Database: OVID Medline Epub Ahead of Print, In-Process & Other Non-Indexed Citations, Ovid MEDLINE(R) Daily and Ovid MEDLINE(R) 1946 to Present

Search Strategy:

--------------------------------------------------------------------------------

1 diabetes mellitus, type 2/ (149514)

2 type 2 diabetes.tw. (142099)

3 *counseling/ or directive counselling/ (17075)

4 coaching.mp. (7871)

5 "one-on-one".tw. (3598)

6 (individual* adj2 counsel*).tw. (2697)

7 ("face to face" not (face-to-face adj2 interview*)).tw. (20705)

8 telemedicine.mp. (39697)

9 *Internet/ (39124)

10 virtual.mp. (75684)

11 exp cell phone/ or telephone?.mp. (86734)

12 (smart phone? or smart phone? or text messag* or app? or computer application? or phone?).tw. (74837)

13 self-management.mp. (23469)

14 1 or 2 (200685)

15 or/3-13 (344444)

16 14 and 15 (5799)

17 limit 16 to english language (5667)

18 ((random* allocat* or randomi#* or clinical trial? or meta-analy* or metaanaly* or (systematic or quantitative or evidence-based)) adj2 (review? or overview?)).tw. (249056)

19 randomized controlled trial.pt. or randomized controlled trial/ or clinical trial/ or clinical trial.pt. or meta-analysis/ or meta-analysis.pt. or review.pt. (3851915)

20 or/18-19 (3933919)

21 17 and 20 (1896)

22 limit 17 to (comment or editorial or letter or news) (98)

23 21 not 22 (1885)

EMBASE/EMCARE

Database: Embase <1974 to 2021 December 03>

Search Strategy:

--------------------------------------------------------------------------------

1 non insulin dependent diabetes mellitus/ (287559)

2 type 2 diabetes.tw. (216122)

3 1 or 2 (327731)

4 *counseling/ or directive counselling/ (17112)

5 coaching.mp. (10818)

6 "one-on-one".tw. (5222)

7 (individual* adj2 counsel*).tw. (3819)

8 ("face to face" not (face-to-face adj2 interview*)).tw. (27965)

9 telemedicine/ or teleconsultation/ or telemonitoring/ (48259)

10 telemedicine.tw. (19728)

11 *Internet/ (36831)

12 virtual.mp. (100998)

13 exp mobile phone/ (36986)

14 (smart phone? or smart phone? or text messag* or app? or computer application? or phone?).tw. (109419)

15 self-management.mp. (32423)

16 or/4-15 (383152)

17 3 and 16 (7493)

18 limit 17 to english language (7325)

19 ((random* allocat* or randomi#* or clinical trial? or meta-analy* or metaanaly* or (systematic or quantitative or evidence-based)) adj2 (review? or overview?)).tw. (305003)

20 exp randomized controlled trial/ or controlled clinical trial/ (874802)

21 exp meta analysis/ (231649)

22 clinical trial/ or clinical study/ or multicenter study/ or phase 3 clinical trial/ (1381066)

23 or/19-22 (2100116)

24 18 and 23 (2022) EMBASE

EMCARE 976

PSYCINFO

Database: APA PsycInfo <1806 to November Week 5 2021>

Search Strategy:

--------------------------------------------------------------------------------

1 exp type 2 diabetes/ (5167)

2 type 2 diabetes.tw. (7884)

3 1 or 2 (9206)

4 *counseling/ (20072)

5 coaching.mp. (13202)

6 "one-on-one".tw. (3875)

7 (individual* adj2 counsel*).tw. (2785)

8 ("face to face" not (face-to-face adj2 interview*)).tw. (16881)

9 telemedicine/ or teleconsultation/ (6313)

10 telemedicine.tw. (2633)

11 *internet/ (23105)

12 electronic communication/ or social media/ or text messaging/ (15697)

13 virtual.mp. (27815)

14 exp mobile phones/ or mobile devices/ or mobile applications/ (9537)

15 (smart phone? or smart phone? or text messag* or app? or computer application? or phone?).tw. (25690)

16 self-management.mp. (12790)

17 or/4-16 (156529)

18 3 and 17 (1489)

19 limit 18 to english language (1439)

20 ((random* allocat* or randomi#* or clinical trial? or meta-analy* or metaanaly* or (systematic or quantitative or evidence-based)) adj2 (review? or overview?)).tw. (46551)

21 exp Randomized Controlled Trials/ or exp Clinical Trials/ (13053)

22 "systematic review"/ or meta analysis/ (5670)

23 or/20-22 (62481)

24 19 and 23 (90)

COCHRANE

Search Name:

Date Run: 05/12/2021 22:41:48

Comment:

ID Search Hits

#1 MeSH descriptor: [Diabetes Mellitus, Type 2] explode all trees 19238

#2 type 2 diabetes 71126

#3 #1 or #2 71127

#4 MeSH descriptor: [Counseling] this term only 4476

#5 MeSH descriptor: [Directive Counseling] this term only 413

#6 coaching 3944

#7 "one-on-one" 1399

#8 (individual* near/2 counsel*) 1609

#9 ("face to face" not ("face-to-face" near/2 interview*)) 7077

#10 MeSH descriptor: [Telemedicine] explode all trees 3080

#11 telemedicine 4926

#12 MeSH descriptor: [Internet] this term only 4057

#13 "virtual care" or virtual consult* or virtual counsel* or virtual monitor* or "virtual medicine" 3089

#14 MeSH descriptor: [Cell Phone] explode all trees 2103

#15 MeSH descriptor: [Telephone] this term only 2280

#16 "smart phone" or "smart phones" or smartphone* or "text messaging" or "text messages" or "text message" or app or apps or "computer application" or "computer applications" or phone or phones 27584

#17 self-management 9081

#18 MeSH descriptor: [Self-Management] explode all trees 612

#19 #4 or #5 or #6 or #7 or #8 or #9 or #10 or #11 or #12 or #13 or #14 or #15 or #16 or #17 or #18 57962

#20 #3 and #19 5321

702 reviews

4458 trials

CINAHL

| Sunday, December 05, 2021 9:09:56 PM | | | |
| --- | --- | --- | --- |
| **#** | **Query** | **Results** |  |
| S23 | S16 AND S21 | 1,027 |  |
| S22 | S16 AND S21 | 1,067 |  |
| S21 | S17 OR S18 OR S19 OR S20 | 550,655 |  |
| S20 | (MH "Meta Analysis") | 58,983 |  |
| S19 | (MH "Systematic Review") | 103,264 |  |
| S18 | ((random* allocat* or randomi* or clinical trial* or meta- analy* or metaanaly* or (systematic or quantitative or evidence-based)) n2 (review* or overview*)) | 251,270 |  |
| S17 | (MH "Randomized Controlled Trials+") OR (MH "Clinical Trials+") | 329,227 |  |
| S16 | S3 AND S15 | 4,391 |  |
| S15 | S4 OR S5 OR S6 OR S7 OR S8 OR S9 OR S10 OR S11 OR S12 OR S13 OR S14 | 180,878 |  |
| S14 | MH self-management OR self-management | 32,736 |  |
| S13 | smart phone* or smart phone* or text messag* or app or apps or computer application* or phone* | 43,629 |  |
| S12 | (MH "Cellular Phone") OR (MH "Text Messaging") OR (MH "Smartphone") OR (MH "Voice Mail") | 8,659 |  |
| S11 | virtual | 32,293 |  |
| S10 | (MM "Internet") | 27,629 |  |
| S9 | telemedicine | 23,271 |  |
| S8 | (MH "Telemedicine") OR (MH "Remote Consultation") OR (MH "Telenursing") | 18,412 |  |
| S7 | ("face to face" not (face-to-face N2 interview*)). | 9,913 |  |
| S6 | (individual* N2 counsel*) | 1,790 |  |
| S5 | "coaching" | 6,532 |  |
| S4 | (MM "Counseling") | 15,113 |  |
| S3 | S1 OR S2 | 83,091 |  |
| S2 | TX type 2 diabetes | 55,545 |  |
| S1 | (MH "Diabetes Mellitus, Type 2") | 66,477 |  |

# Characteristics of Included Studies

Balducci

| Country, Year | Italy, 2019 |
| --- | --- |
| Question/Study Objective | To investigate whether a behavioral intervention strategy can produce a sustained increase in physical activity and reduction in sedentary time among individuals with type 2 diabetes |
| Study Design | Open-label, assessor-blinded, randomized clinical superiority trial |
| Inclusion/Exclusion Criteria | Inclusion: type 2 diabetes (defined by the American Diabetes Association criteria) for at least 1 year, age 40 to 80 years, body mass index of 27 to 40, physical inactivity (ie, insufficient amounts of physical activity according to current guidelines), sedentary lifestyle (ie, >8 hours of time awake spent in a sitting or reclining posture) for at least 6 months, ability to walk 1.6 km without assistance, and clearance by a cardiologist.  Exclusion: NR |
| Sample Size | O: 300, I: 150, C: 150 |
| Loss to Follow-Up | n (%) - O: 33 (11), I: 17 (11.3); C: 16 (10.7) |
| Age | Mean (SD) - I: 61.0 (9.7), C: 62.3 (10.1) |
| Gender | n (%) - Female O: 116 (38.7); I: 59 (39.3); C: 57 (38.0); Male O: 184 (61.3); I: 91 (60.7); C: 93 (62.0) |
| Race/Ethnicity | NR |
| BMI | I: 30.0 (4.9); C: 30.1 (5.3) |
| Duration of Diabetes | Median (IQR) I: 8.0 (4.0-16.0); C: 9.0 (4.0-15.0) |
| Baseline A1C% | Mean (SD) I: 7.4 (1.6); C: 7.3 (1.4) |
| Description of Intervention | Participants in the behavioral intervention group participated in 1 individual theoretical counseling session, conducted by a diabetologist, and 8 biweekly individual theoretical and practical counseling sessions, conducted by a certified exercise specialist, per year for 3 years. |
| Who Delivered Intervention | Certified exercise specialist/diabetologist |
| Description of Coaching Interactions | Frequency: 1 theoretical counselling session, 8 biweekly individual theoretical and practical counselling sessions per year  Duration: NR  Mode: Individual face to face sessions |
| Location/Site of Delivery | Outpatient diabetes clinics |
| Description of Control | Participants in the standard care group received only general physician recommendations for increasing daily physical activity and decreasing sedentary time. |
| Duration of Intervention | I: 3 years; C: 3 years |
| Length of Follow-Up Beyond Post-Intervention | NA |
| Serious Adverse Events | I: 41; C: 59 (hypoglycemia, tachycardia/arrhythmia, musculoskeletal injury/discomfort) |
| Funding Source | Metabolic Fitness Association, Monterotondo, Rome, Italy |

Cummings

| Country, Year | U.S.A., 2019 |
| --- | --- |
| Question/Study Objective | To evaluate the effect of cognitive behavioral therapy (CBT) plus lifestyle counseling in primary care on hemoglobin A1c (HbA1c) in rural adult patients with type 2 diabetes (T2D) and comorbid depressive or regimen-related distress (RRD) symptoms |
| Study Design | Randomized controlled trial |
| Inclusion/Exclusion Criteria | Inclusion: adult patients (18–75 years) with a medical record–established history of T2D with an HbA1c at screening >7.0% (53 mmol/mol) and with a positive screen for symptoms of distress using the Diabetes Distress Scale 2 (DDS-2) item screener (positive = mean score > or = 3 on DDS-2) and/or a positive screen for symptoms of depression on the Patient Health Questionnaire 2 (PHQ-2) item screener (positive = total score > or = 3 on PHQ-2).  Exclusion: Exclusion criteria for screening included a medical record–established diagnosis of advanced disease (e.g., end-stage renal disease, advanced heart failure, blindness, or metastatic cancer) or the presence of alcoholism, cognitive impairment, or major psychiatric illness that would preclude active participation.  If the patient signed consent and met all the screening criteria described above, including having an HbA1c value that day that was >7.0%, then he/she was scheduled for an enrollment visit. |
| Sample Size | O: 139; I: 67; C: 72 |
| Loss to Follow-Up | n (%) - 19 (13.7); I: 10 (13.9); C: 9 (13.4) |
| Age | Mean (SD) – O: 52.6 (9.6); I: 51.0 (9.0); C: 53.0 (9.0) |
| Gender | n (%) – Female O: 108 (77.7); I: 53 (79); C: 55 (76); Male O: 31 (22.3); I: 14 (21); C: 17 (24) |
| Race/Ethnicity | % African American - I: 77%; C: 67% |
| BMI | Weight (lb) Mean (SD) - O: 225 (56.9); I: 217 (57); C: 232 (56) |
| Duration of Diabetes | NR |
| Baseline A1C% | Mean - O: 81; I: 84; C: 79 |
| Description of Intervention | The small-changes lifestyle treatment subgroup included intervention arm patients with low levels of diabetes-related distress and/or depressive symptoms, and the nurse care manager delivered a twice-monthly telephonic intervention focused on lifestyle modifications to improve diabetes and mood based on the small-changes health behavior change model that we have previously described in detail.  The CBT subgroup intervention focused on the reduction of depressive and/or RRD symptoms through modification of negative thoughts and problematic behaviors as well as improvement of diabetes self-management strategies. Sessions were delivered by a clinical health psychologist as well as a doctoral student in clinical health psychology. CBT intervention components were guided by two evidence-based treatment manuals for behavioral activation. Session content used cognitive techniques to identify and challenge general and diabetes-specific cognitive distortions that result in maladaptive behavior, in combination with behavioral techniques, including behavioral activation and specific behavior change strategies related to diabetes and/or mood (self-monitoring, sleep hygiene, eating habits, etc.). These sessions occurred face to face (in the primary care clinic) or via telephonic visits with the health psychologist. Patients who met criteria for the CBT subgroup intervention and yet had more intermediate concerns were provided PST. The PST intervention was based on PST for primary care, which is an adapted version of PST specifically for use in primary care clinics. PST, a variant of CBT, focuses on the facilitation of effective coping and adaptive problem-solving skills and has been shown to be an effective intervention strategy for improvement in diabetes-specific outcomes.  Standard medical care was continued for both arms. However, primary care providers for patients in the intervention arm were offered consultation with a diabetologist to optimize medical management. Primary care providers were asked to titrate medications to appropriate therapeutic dosages based on finger-stick blood glucose response and subsequent HbA1c values. Patient response, adherence, and potential for  side effects were monitored approximately quarterly by the nurse care manager during face-to-face and telephone follow-up, with particular attention to the potential for hypoglycemia associated with insulin and sulfonylurea drugs.  All intervention patients had access to a trained community health worker (CHW) who had extensive experience promoting healthy behaviors for chronic disease management in the targeted region. This CHW provided quarterly telephonic peer support and served as a navigator to community resources that helped patients address logistical challenges to implementing healthy behaviors, problem solving, and accessing healthy food/activity in the target community. |
| Who Delivered Intervention | A team of trained behavioral providers working together, including a nurse care manager who provided small changes lifestyle coaching, a psychologist and clinical health psychology doctoral student who provided CBT sessions including elements of problem-solving therapy (PST), where indicated, and a community health worker (CHW) who provided navigation and social support. |
| Description of Coaching Interactions | Frequency: 1 individual orientation session, 12 individually tailed behavioural treatment sessions  Duration: 30 to 60 minutes  Mode: In-person or optional telephone |
| Location/Site of Delivery | A large academic family medicine practice in the southeastern U.S. |
| Description of Control | Standard medical care |
| Duration of Intervention | I: 12 months; C: 12 months |
| Length of Follow-Up Beyond Post-Intervention | NA |
| Serious Adverse Events | NR |
| Funding Source | East Carolina University |

Jutterström

| Country, Year | Sweden, 2016 |
| --- | --- |
| Question/Study Objective | To evaluate the effect of a patient-centered self-management support, in type 2 diabetes (T2D) with regard to metabolic changes |
| Study Design | Randomized controlled trial with three arms and external control group |
| Inclusion/Exclusion Criteria | Inclusion: diagnosed with T2D within three years, aged 40–80 years, Swedish speaking, and no diagnosed cognitive impairment or other severe illnesses. They had not received patient education other than information given to newly diagnosed T2D patients. |
| Sample Size | O: 327; I: 35; IC: 36; EC: 54 |
| Loss to Follow-Up | n (%) O: 23 (8.5); I: 2 (5.7); IC: 4 (11.1); EC: 7 (13.0) |
| Age | Mean (SD) O: 64.5 (9.58); I: 64.9 (11.10); IC: 62.6 (10.61); EC: 66.2 (8.75) |
| Gender | n (%) Female O: 68 (37.4); I: 13 (38.2); IC: 16 (47.1); EC: 19 (37.3); Male: O: 114 (62.6) I: 21 (61.8); IC: 18 (52.9); EC: 32 (62.7) |
| Race/Ethnicity | NR |
| BMI | O: 30.40 (5.45); I: 31.76 (5.73); IC: 30.56 (5.81); EC: 29.62 (5.27) |
| Duration of Diabetes | NR |
| Baseline A1C% | Mean (SD) O: 5.78 (0.88); I: 5.8 (0.87); IC: 5.8 (0.77); EC: 5.5 (0.84) |
| Description of Intervention | The three groups were group intervention (GI), individual intervention (II), or internal control (IC). All three groups of patients were cared for by the same diabetes nurse at each HCC. Patients randomized to any of the three groups were listed by random and got a number 1, 2, 3, 4, 5, etc.; subsequently they were invited by letter and telephone. The procedure started with an invitation to the first 15 patients in each group and from each HCC.  In the patient intervention, participants in the GI and II groups were invited to six sessions of 45–90 min each, over a period of up to six months. In the GI groups, the patients reflected aspects of living with type 2 diabetes together and the DSNs acted as a moderator, while she acted as the reflective part in the II groups. The content of the intervention is described below. Together with the DSNs, the participants decided when and how often the six sessions should take place, and the intervention period thereby varied between 2 and 6 months. The intervention consisted of either discussions in groups or patients or individual conversations with the DSN, depending on the arm of allocation. During the six sessions, the participants were free to discuss issues they considered important in relation to their experiences with the disease. Each session also had a theme; their views of the image of the illness; the meaning of the diagnosis; the illness integration over time; time and the space for management of the illness in daily life; views on the responsibilities of self-management; and lastly, their prospects for living life with an illness such as medical facts were conveyed only when requested by the participants. The II participants met the local diabetes nurse one-on-one, while the GI participants met in groups where the diabetes nurse acted as a moderator who made sure that everyone in the group participated in the discussion. |
| Who Delivered Intervention | Nine of the nurses had a university education in diabetes care while the tenth had completed diabetes courses offered by pharmaceutical companies. All DSNs participated in a preparatory workshop before the intervention. |
| Description of Coaching Interactions | Frequency: 6 sessions for up to 6 months  Duration: 45-90 minutes  Mode: Face to face |
| Location/Site of Delivery | 9 health care centers in country councils in Northern Sweden |
| Description of Control | An external control group (EC) from an HCC in another county council was recruited with the same inclusion criteria and a similar process for inclusion was used where all patients were listed and one-third of the patients were included. The reason for having an external control group was to control for spillover effects, i.e., unintended positive consequences, which in this case meant that the nurses, when learning a new approach, could also adopt and use the new knowledge on other patients outside the intervention group.  Patients randomized to control groups (IC, EC) received standard care, which normally included 1–2 visits per year according to national guidelines. |
| Duration of Intervention | I: 2-6 months; C: 12 months |
| Length of Follow-Up Beyond Post-Intervention | 5-year study period |
| Serious Adverse Events | NR |
| Funding Source | Strategic Research Programme in Care Sciences, Umeå University and Karolinska Institute, and the Swedish Diabetes Association, the County Council of Västerbotten and Umeå University |

Karhula

| Country, Year | Finland, 2015 |
| --- | --- |
| Question/Study Objective | To study whether a structured mobile phone-based health coaching program, which was supported by a remote monitoring system, could be used to improve the health-related quality of life (HRQL) and/or the clinical measures of type 2 diabetes and heart disease patients |
| Study Design | Randomized controlled trial |
| Inclusion/Exclusion Criteria | Inclusion: diagnosis of type 2 diabetes, glycosylated hemoglobin (HbA1c) level, which needed to be above 6.5% within 1 year prior to the screening, diagnosed with diabetes at least 3 months earlier, 18 years of age or older, ability to fill in questionnaires in Finnish, ability to use the RPM system and the devices provided, having adequate cognitive capacities to participate, being able to walk  Exclusion: NR |
| Sample Size | O: 287; I: 208; C: 79 |
| Loss to Follow-Up | n (%) O: 62 (21.6); I: 46 (22.1); C: 16 (20.3) |
| Age | Mean (SD) – I: 66.6 (8.2); C: 65.5 (9.6) |
| Gender | n (%) - Female O: 111 (44.4); I: 81 (45); C: 30 (43), Male O: 139 (55.6); I: 99 (55); C: 40 (57) |
| Race/Ethnicity | NR |
| BMI | Mean (SD) – I: 31.1 (5.4); C: 30.9 (5.7) |
| Duration of Diabetes | NR |
| Baseline A1C% | Median - I: 7.25; C: 7.20 |
| Description of Intervention | The intervention consisted of health coaching over mobile phones and self-monitoring of health parameters with the help of a remote patient monitoring (RPM) system. A comprehensive evaluation of the patient’s clinical, mental, and social condition was made during the first coaching call and small, achievable health behavior changes were agreed upon with the patient. A self-management plan was created based on the targeted changes. During the mobile phone calls, the health coach provided information, assistance, and support to the patients. The health coaching approach was provided by Pfizer Oy. The approach followed Wagner’s Chronic Care Model—one of the key foundational constructs for the approach of chronic care management—and has been developed and tested earlier. |
| Who Delivered Intervention | Personal health coach trained to obtain the needed knowledge about Pfizer’s health coaching model, behavioral management skills, remote monitoring system, and trial procedures |
| Description of Coaching Interactions | Frequency: Every 4-6 weeks  Duration: 30 minutes  Mode: Telephone |
| Location/Site of Delivery | South Karelia Social and Health Care District (Eksote) |
| Description of Control | Patients assigned to the control group received the care they would have received in the absence of the study. As part of standard care, patients suffering from type 2 diabetes receive a disease management information booklet at the time of diagnosis. Standard care includes laboratory tests taken once a year and 1 appointment or phone call by a nurse or doctor. Patients can contact health care services any time they feel they need to. |
| Duration of Intervention | I: 12 months; C: 12 months |
| Length of Follow-Up Beyond Post-Intervention | NA |
| Serious Adverse Events | NR |
| Funding Source | 50% of the funding of this study was received from the European Commission Information and Communication Technologies Policy Support Program (ICT PSP) 2009 of the Competitiveness and Innovation framework Programme (CIP), as part of the Renewing Health Project involving nine European countries. The other 50% of the funding was provided by Eksote. |

Naik

| Country, Year | U.S.A., 2019 |
| --- | --- |
| Question/Study Objective | To evaluate the effectiveness of proactive population screening plus telephone delivery of a collaborative goal-setting intervention among high-risk patients with uncontrolled diabetes and depression |
| Study Design | Randomized clinical trial |
| Inclusion/Exclusion Criteria | Inclusion: Veterans with uncontrolled diabetes (defined by International Classification of Diseases, Ninth Revision diagnosis code 250.XX and HbA1c of > or =7.5% for 1 year before the study) who lived at least 20 miles from the main Veterans Health Administration hospital in Houston, Texas, or who received primary care services within a MEDVAMC satellite community-based clinic across Southeast Texas.  Exclusion: If there was an absence of depression symptoms, a telephone-based coaching intervention would be inappropriate (eg, the patient had severe cognitive impairment or mental health condition, hearing or visual impairment, or active suicidal ideation), or presence of significant hypoglycemic events or substance abuse. |
| Sample Size | O: 225; I: 136; C: 89 |
| Loss to Follow-Up | n (%) – O: 59 (26.2); I: 40 (29.4); C: 19 (21.3) |
| Age | Mean (SD) – O: 61.9 (8.3) |
| Gender | n (%) – Female O:23 (10.2); I: 15 (11); C: 8 (9); Male O: 202 (89.8); I: 121 (89); C: 81 (91) |
| Race/Ethnicity | n (%) - White - I: 73 (53.7); C: 51 (57.3); non-Hispanic black - I: 41 (30.1); C: 16 (18.0); Hispanic - I: 12 (8.8); C: 11 (12.4); Other - I: 10 (7.4); C: 11 (12.4) |
| BMI | NR |
| Duration of Diabetes | NR |
| Baseline A1C% | Mean (SD) – O: 9.3 (1.4); I: 9.2 (1.4); C: 9.3 (1.5) |
| Description of Intervention | During the first 2 patient sessions, HOPE coaches focused on building rapport, introducing and clarifying values, collaboratively setting initial goals, identifying potential skill sets to address goals, and empowering patients to advocate for their health through active communication with their clinicians. For sessions 3 through 6, participants focused on discrete skill modules (increasing pleasant activities, using thoughts to improve wellness, diet, physical activity, medication management, and relaxation) customized to meet their diabetes and depression goals. Sessions 7 through 9 focused on maintenance skills (reviewing action plans and overcoming barriers). Skills emphasized in the modules were designed to improve diabetes- and depression-related outcomes simultaneously. The HOPE modules stressed the importance of the coach-patient relationship as critical to improvement in participant physical and/or emotional self-management. During months 7 to 12, participants received usual primary care without contact from HOPE coaches. |
| Who Delivered Intervention | Twenty-four trained health professionals or coaches (18 female) included psychologists (n = 16), nurses (n = 5), pharmacists (n = 2), and social workers (n = 1). Most (n = 18) were at the MEDVAMC; 6 were at a Veterans Health Administration community-based clinic |
| Description of Coaching Interactions | Frequency: 9 sessions biweekly from months 1 to 3 and monthly from months 4 to 6; no contact months 7 to 12  Duration: 30-40 minutes from months 1 to 3 and 15 minutes from month 4 to 6  Mode: Telephone |
| Location/Site of Delivery | MEDVAMC and 6 affiliated community-based outpatient clinics across Southeast Texas |
| Description of Control | In addition to usual care, EUC participants were informed about their high-risk status (uncontrolled diabetes status and clinically significant depression symptoms) and were given related educational materials. Study assessments were conducted for EUC participants via telephone, and educational materials were mailed. Participants were encouraged to address these results with their primary care clinician. |
| Duration of Intervention | I: 6 months; C: 6 months |
| Length of Follow-Up Beyond Post-Intervention | 12 months |
| Serious Adverse Events | NR |
| Funding Source | Grant 10-135 from the Veterans Health Administration Health Services Research and Development Office (Drs Cully and Naik) and by grant K23DK11034 from the National Institute of Diabetes and Digestive and Kidney Diseases, National Institutes of Health (Dr Vaughan). |

Odnoletkova

| Country, Year | Belgium, 2016 |
| --- | --- |
| Question/Study Objective | To investigate the effect of the COACH programme on HbA1c and other modifiable diabetes risk factors in people with Type 2 diabetes in a primary care setting in Belgium compared with usual care |
| Study Design | Parallel group, randomized controlled trial |
| Inclusion/Exclusion Criteria | Inclusion: people April 2012 and June 2013, 3115 people were invited to participate in the study. Study participants were adults aged 18–75 years with a diagnosis of Type 2 diabetes, who were receiving glycose-lowering oral and/or injectable therapy.  Exclusion: included corticoid therapy and/or a debilitating coexisting medical condition, such as dialysis, mental illness or cancer; residence in long-term care facilities; pregnancy; and insufficient proficiency in Dutch. |
| Sample Size | O: 3115; I: 287; C: 287 |
| Loss to Follow-Up | n (%) - O: 62 (11); I: 35 (12); C: 27 (9) |
| Age | O: Median of 64 years; I: Mean (SD) 63.8 (8.7); C: Mean (SD) 62.4 (8.9) |
| Gender | Female - O: 221 (38.5); I: 114 (40); C: 107 (37); Male – O: 353 (61.5); I: 173 (60); C: 180 (63) |
| Race/Ethnicity | NR |
| BMI | Mean (SD) - O: 30 (5); I: 30.2 (4.9); C: 30.6 (5.2) |
| Duration of Diabetes | Years (SD) - O: 7 years; I: equal or less than 2 years = 46 (16%); equal or greater 10 years = 94 (33%); C: equal or less than 2 years = 41 (14%); equal or greater 10 years = 91 (32%) |
| Baseline A1C% | Mean (SD) - O: 53 (11); I: 53 (12); C: 53 (11) |
| Description of Intervention | The COACH programme is designed to empower patients to take responsibility for the achievement of their risk factor targets. The coach identifies the ‘treatment gaps’ in the management of each diabetes risk factor, i.e., failure to achieve the guideline-recommended goals, and helps the patient to identify strategies to close the treatment gap, including lifestyle adjustments and adherence to recommended medication therapy. The underlying ‘COACH model’ is a continuous quality improvement cycle, which includes bridging the knowledge gap, assertiveness training, setting an action plan and (re)assessment. The programme consisted of five telephone sessions of a mean (range) duration of 30 (10–45) min, delivered at a mean (range) interval of 5 (3–8) weeks by a certified diabetes nurse educator (hereafter referred to as the ‘coach’) after a 5-day training course. It consisted of an update of the best practice guidelines for the management of Type 2 diabetes, motivational interviewing techniques and software program use. All coaches were employed by a Flemish home care organization, ‘Solidariteit voor het Gezin’. The intervention group received a welcome package containing a nutrition guide, waist circumference metre, BMI calculator and a set for self-monitoring of blood glucose. Participants were instructed on how to perform self-monitoring of blood glucose and interpret the results and were advised on the measurement frequency. They were encouraged to perform the necessary check-ups and to discuss with their general practitioner (GP) drug treatment intensification when appropriate. The coaches analysed patient risk profiles based on the baseline assessment data and consulted GPs on the individual therapeutic goals before the start of the programme. After each session, a written coaching report was prepared by the coach and sent to the participant and his/her GP. The reports contained a comparison between the recommended and the actual outcomes for diabetes risk factors and an agreed action plan to bridge any resulting gap.  The intervention quality control measurements included a review of coaching reports by one of the present authors (I.O.) during the first 3 months and selectively thereafter, audio-recording of several sessions, and weekly programme monitoring briefings. |
| Who Delivered Intervention | Certified diabetes nurse educators after additional training |
| Description of Coaching Interactions | Frequency: 5 monthly telephone sessions  Duration: 30 minutes on average  Mode: Telephone |
| Location/Site of Delivery | Primary care settings |
| Description of Control | The control group received usual care. In Belgium, patients on oral glycaemia-lowering drugs are predominantly treated by their GPs. When insulin therapy needs to be initiated, patients are entitled to a ‘diabetes care trajectory’ that includes diabetes education by a certified diabetes educator and an annual consultation with an endocrinologist, in addition to the regular GP visits. People with advanced diabetes, in need of three or more insulin injections per day, are normally treated by an endocrinologist-led hospital-based diabetes team. All study participants received a DVD with educational material on Type 2 diabetes. The laboratory results of the blood analysis were mailed to all study participants and their GPs. |
| Duration of Intervention | I: 6 months; C: 6 months |
| Length of Follow-Up Beyond Post-Intervention | 18 months |
| Serious Adverse Events | NR |
| Funding Source | The European Regional Development Fund and the Flemish Government. Partena, MSD and Abbott provided a scientific grant for the clinical trial. |

Sherifali

| Country, Year | Canada, 2021 |
| --- | --- |
| Question/Study Objective | To evaluate the effect of a 12-month telephone diabetes health coaching (DHC) intervention on glycemic control in persons living with T2DM |
| Study Design | Community-based randomized controlled trial |
| Inclusion/Exclusion Criteria | Inclusion: > or =18 years of age; b) a T2DM diagnosis (any duration); c) an A1C level >7.5% within 6 months before randomization; d) ability to read and write in English; and e) telephone access  Exclusion: a) pregnancy, b) debilitating coexisting conditions (i.e., mental illness, impaired cognition) and c) underlying medical conditions that could provide misleading A1C levels |
| Sample Size | O: 365; I: 188; C: 177 |
| Loss to Follow-Up | n (%) – O: 8 (2.2%); I: 6 (3.4%); C: 2 (1.1%) |
| Age | Mean (SD) – I: 56.82 (11.69); C: 59.05 (11.79) |
| Gender | n (%) – Female O: 183 (50.1); I: 89 (47.34); C: 94 (53.11); Male O: 182 (49.9); I: 99 (52.66); C: 83 (46.89) |
| Race/Ethnicity | Caucasian % - I: 150 (79.79); C: 144 (81.36) |
| BMI | Mean (SD) – I: 34.71 (7.80); C: 35.36 (8.35) |
| Duration of Diabetes | Years (SD) – I: 10.07 (9.08); C: 9.31 (7.99) |
| Baseline A1C% | Mean (SD) – I: 9.10 (1.65); C: 8.86 (1.50) |
| Description of Intervention | The topic or agenda of each telephone call was determined by the participant or as agreed upon from the previous coaching session. The diabetes health coaching intervention comprised an evidence-informed model of care that included: 1) case management and monitoring; 2) diabetes self-management education and support; 3) behaviour modification, goal setting and reinforcement; and 4) general psychosocial support. The diabetes coaching model philosophy was to provide flexibility and personalization, recognizing that all 4 components are necessary for diabetes self-management and that each component may be required in different amounts and at different times, depending on each participant’s circumstances, goals and needs. |
| Who Delivered Intervention | Registered nurse/certified diabetes educator with training in the DHC model, motivational interviewing and behavioural design, specifically a certification in Tiny Habits coaching, which emphasizes small, positive habits customized to one’s environment, ability and motivation |
| Description of Coaching Interactions | Frequency: Weekly for first 6 months, monthly for last 6 months  Duration: 15 minutes  Mode: Telephone |
| Location/Site of Delivery | Waterloo region of Ontario, Canada |
| Description of Control | All participants in the study received access to usual diabetes education (individual or group) provided by nurses and/or dietitians, typically every 3 to 6 months, along with community resources and an accelerometer. |
| Duration of Intervention | I: 12 months; C: 12 months |
| Length of Follow-Up Beyond Post-Intervention | NA |
| Serious Adverse Events | NR |
| Funding Source | Canadian Institutes of Health Research, Canada (Grant No. 311588) |

Varney

| Country, Year | Australia, 2014 |
| --- | --- |
| Question/Study Objective | To measure the effect of a 6-month telephone coaching intervention on glycaemic control, risk factor status and adherence to diabetes management practices at the intervention’s conclusion (6 months) and at 12 months |
| Study Design | Randomized controlled trial |
| Inclusion/Exclusion Criteria | Inclusion: adults with T2DM and HbA1C >7%  Exclusion: unable to provide consent, non-English speaking, cognitively impaired, receiving palliative care, severely hearing impaired or without telephone access |
| Sample Size | O: 94; I: 47; C: 47 |
| Loss to Follow-Up | O: 6 months: 81 (86.2), 12 months: 71 (75.5); I: 6 months: 9 (19.1), 12 months: 12 (25.5); C: 6 months: 4 (8.5), 12 months: 11 (23.4) |
| Age | Mean (95% CI) - I: 59 (56-62); C: 64 (61-66) |
| Gender | n (%) - Female O: 30 (32.0); I: 13 (28); C: 17 (36), Male O: 64 (68.0); I: 34 (72); C: 30 (64) |
| Race/Ethnicity | n (%) - Caucasian I: 46 (98); C: 37 (79); Asian/Indian I: 1 (2); C: 8 (17); Afro-Caribbean I: 0 (0); C: 2 (4) |
| BMI | Mean (95% CI) - I: 32.1 (30.3-33.9); C: 30.9 (29.1-32.6) |
| Duration of Diabetes | Years (SD) – I: 12.6 (10.2-15.0); C: 13.1 (10.7-15.6) |
| Baseline A1C% | I: 8.2 (8.0-9.7); C: 8.5 (8.1-8.9) |
| Description of Intervention | In addition to usual diabetes care, intervention group participants received 6 months of telephone coaching. Advice given in coaching sessions was consistent with Australian guidelines. During initial coaching sessions, a diet history was taken. Participants were encouraged to follow a low saturated fat, high-fibre diet, with 50% of energy from carbohydrates, and were encouraged to exercise for 150 min per week. Risk factor status and adherence to monitoring requirements were based on information collected at baseline. For treatment goals and risk factors not at target levels, the dietary, lifestyle and medication changes required to improve these parameters were discussed. The coach delivering the intervention did not prescribe medication, therefore, participants were advised to discuss medication changes with their general practitioner (GP). Discrepancies between participants’ adherence to self-care activities (diet and physical activity) and monitoring requirements (foot checks, eye checks and vaccinations) were highlighted and the appropriate management schedule was explained. Participant goals were then agreed, be this a change in diet or a podiatry appointment for an overdue  foot examination. Following each coaching session, the participant and their GP received a letter summarising the participant’s goals.  During subsequent coaching sessions, progress towards treatment goals, risk factor status, adherence to self-care and monitoring requirements were reassessed. If goals were not achieved, barriers to goal attainment were identified, an action plan addressing these barriers was agreed and new goals were established. This process was repeated throughout the intervention. |
| Who Delivered Intervention | Dietitian with experience in cardiovascular disease and T2D |
| Description of Coaching Interactions | Frequency: Monthly, mean number of sessions 6.0 (range 4-9)  Duration: Flexible, determined by time required to establish participant goals. Typically, initial and follow-up sessions took 45 and 20 minutes respectively.  Mode: Telephone |
| Location/Site of Delivery | Diabetes Clinic of St Vincent’s Hospital Melbourne (STV), an Australian public teaching hospital |
| Description of Control | Controls did not receive the telephone coaching intervention, or any contact from the researchers, with the exception of telephone calls to arrange baseline, 6- and 12-month assessment appointments. Control group participants could access STV usual care services, including a diabetes clinic staffed by endocrinologists, diabetes educators and dietitians. STV patients typically attend the diabetes clinic 3–6 monthly, with GP visits occurring at the patient’s discretion. |
| Duration of Intervention | I: 6 months; C: 6 months |
| Length of Follow-Up Beyond Post-Intervention | 12 months |
| Serious Adverse Events | NR |
| Funding Source | St Vincent’s Hospital, Research Endowment Fund |

Young

| Country, Year | U.S.A., 2020 |
| --- | --- |
| Question/Study Objective | To evaluate the effectiveness of a nurse coaching program using motivational interviewing paired with mobile health (mHealth) technology on diabetes self-efficacy and self-management for persons with type 2 diabetes |
| Study Design | Randomized controlled trial |
| Inclusion/Exclusion Criteria | Inclusion: (1) aged 18 years or above, (2) receiving care at 1 of the 3 clinics, (3) living with type 2 diabetes and having HbA1c of 6.5% (48 mmol/mol) or higher, and (4) able to speak English  Exclusion: no access to a telephone, were not able to consent because of cognitive impairment, or were pregnant |
| Sample Size | O: 319 (392 invited); I: 158; C: 161 |
| Loss to Follow-Up | n (%) – O: 32 (10.0); I: 26 (16.5); C: 6 (3.7) |
| Age | Mean (SD) – O: 59.07 (11.4); I: 58.96 (11.3); C: 59.18 (11.5) |
| Gender | n (%) – Female O: 148 (47.3); I: 73 (47.4); C: 75 (47.2); Male O: 165 (52.7); I: 81 (52.6); C: 84 (52.8) |
| Race/Ethnicity | Race n (%) - Caucasian I: 96 (63.2); C: 100 (62.9), African American I: 21 (13.8); C: 18 (11.3), Asian I: 11 (7.2); C: 16 (10.1), Other I: 16 (10.5); C: 14 (8.8), More than 1 race I: 8 (5.3); C: 11 (6.9)  Ethnicity n (%) - Hispanic or Latino I: 24 (17.5); C: 18 (12.9), Not Hispanic or Latino I: 113 (82.5); C: 122 (87.1) |
| BMI | NR |
| Duration of Diabetes | NR |
| Baseline A1C% | NR |
| Description of Intervention | We paired each participant with a nurse health coach who delivered 6 individual sessions using a counseling style based on the concepts of MI. Sessions were structured to promote mutual goal setting, enhance self-efficacy in health behaviour change, and assist individuals to derive meaning from the data to reinforce choices and behaviours. Two RN researchers with nurse coaching experience in diabetes audited 8 of the 158 (5%) of the participant sessions and scored the coach using the MITI. They provided timely feedback to the coaches during weekly debriefing sessions, reviewed scores, and discussed optimization strategies by reviewing scenarios.  The initial MI session elicited motivations and set goals with tracking metrics to gauge the progress toward goals at subsequent sessions. Throughout the sessions, the coaches encouraged the participants to identify facilitators and barriers to achieving their health goals. |
| Who Delivered Intervention | Nurse health coaches for the intervention were 3 registered nurses (RNs) with experience in both health coaching and management of chronic disease |
| Description of Coaching Interactions | Frequency: Every 2 weeks for 3 months  Duration: NR  Mode: In-person orientation followed by telephone sessions |
| Location/Site of Delivery | Primary care clinics in Northern California |
| Description of Control | Participants in this group received usual care through their primary care clinic. Usual care comprised standard health care visits with providers and access to classes, resources, and services (i.e., diabetes management and weight loss education, electronic learning videos, and care coordination). At the orientation meeting, the study team members provided instruction on how to access these resources and services as well as how to use the health system’s patient portal (MyChart). |
| Duration of Intervention | I: 3 months; C: 3 months |
| Length of Follow-Up Beyond Post-Intervention | 9 months |
| Serious Adverse Events | NR |
| Funding Source | Patient-Centered Outcomes Research Institute: IHS-1310-07894 |

# TIDieR Components for Included Studies

|  | Name | Why | What | | Who | How | Where | When and How much | Tailoring | Modification | How well | |
| --- | --- | --- | --- | --- | --- | --- | --- | --- | --- | --- | --- | --- |
| Author, year |  |  | Materials | Procedures |  |  |  |  |  |  | Planned | Actual |
| Balducci 2019 | X | X | N/R | X | X | X | X | X | N/R | X | N/R | N/R |
| Cummings 2019 | X | X | X | X | X | X | X | X | X | N/R | N/R | N/R |
| Jutterstrӧm 2016 | X | X | N/R | X | X | X | X | X | X | N/R | N/R | N/R |
| Karhula 2015 | X | X | X | X | X | X | N/R | X | X | X | X | N/R |
| Naik 2019 | X | X | X | X | X | X | X | X | X | N/R | N/R | N/R |
| Odnoletkova 2016 | X | X | X | X | X | X | X | X | X | N/R | X | X |
| Sherifali 2021 | X | X | X | X | X | X | X | X | X | N/R | X | N/R |
| Varney 2014 | X | X | N/R | X | X | X | X | X | X | N/R | N/R | N/R |
| Young 2020 | X | X | X | X | X | X | X | X | X | X | X | N/R |

The characteristics of the studies were evaluated using the Template for Intervention Description and Replication (TIDieR) checklist

| Balducci et al, 2019 | | |
| --- | --- | --- |
| **TIDieR Tool Item** | **Main Paper** | **Other Paper(s)** |
| **BRIEF NAME**  Provide the name or a phrase that describes the intervention. | The Italian Diabetes and Exercise Study 2 behavioral intervention |  |
| **WHY**  Describe any rationale, theory, or goal of the elements essential to the intervention. | The Italian Diabetes and Exercise Study 2 (IDES_2) investigated whether a behavioral intervention strategy was more effective than standard care in promoting a sustained increase in physical activity and reduction in sedentary time in individuals with type 2 diabetes. | SCT and HBM  Trial protocol page 14 |
| **WHAT**  Materials: Describe any physical or informational materials used in the intervention, including those provided to participants or used in intervention delivery or in training of intervention providers.  Provide information on where the materials can be accessed (e.g., online appendix, URL). | N/R |  |
| Procedures: Describe each of the procedures, activities, and/or processes used in the intervention, including any enabling or support activities. | Participants in the behavioral intervention group participated in 1 individual theoretical counseling session, conducted by a diabetologist, and 8 biweekly individual theoretical and practical counseling sessions, conducted by a certified exercise specialist, per year for 3 years. This approach, derived from the original IDES protocol, was conceived to promote a 2-step behavior change to decrease sedentary time by substituting it with a wide range of light-intensity physical activities and/or interrupting prolonged sitting with brief bouts of light-intensity physical activity and to reallocate sedentary time and/or light-intensity physical activity toward gradually increasing amounts of purposeful moderate- to vigorous intensity physical activity.  Participants in the standard care group received only general physician recommendations for increasing daily physical activity and decreasing sedentary time.  Patients from both groups received the same treatment regimen, including dietary prescription, to achieve glycemic, lipid, blood pressure, and body weight targets, according to contemporaneous American Diabetes Association guidelines. Treatment regimens were adjusted at each visit using a prespecified algorithm. |  |
| **WHO PROVIDED**  For each category of intervention provider (e.g., psychologist, nursing assistant), describe their expertise, background and any specific training given. | Physicians and Exercise Specialists. A specific strategy was implemented to train physicians and exercise specialists participating in this trial to standardize procedures, improve the efficacy and safety of the intervention, promote patient adherence, and minimize dropout, as previously detailed. | Trial protocol page 10 |
| **HOW**  Describe the modes of delivery (e.g., face-to-face or by some other mechanism, such as internet or telephone) of the intervention and whether it was provided individually or in a group. | Individually, in person, face to face |  |
| **WHERE**  Describe the type(s) of location(s) where the intervention occurred, including any necessary infrastructure or relevant features. |  | Diabetes Clinic and three specialized gym facilities connected with one of the three Diabetes Clinics  Trial protocol page 15 and 16 |
| **WHEN and HOW MUCH**  Describe the number of times the intervention was delivered and over what period of time including the number of sessions, their schedule, and their duration, intensity or dose. |  | Trial protocol page 11-15  One 30-min session once a year for three years  Eight 75-min sessions once a year for three years - held twice a week |
| **TAILORING**  If an intervention was planned to be personalised, titrated or adapted, then describe what, why, when, and how. | N/R |  |
| **MODIFICATION**  If an intervention was modified during the course of the study, describe the changes (what, why, when, and how). | This approach, derived from the original IDES protocol, was conceived to promote a 2-step behavior change to decrease sedentary time by substituting it with a wide range of light-intensity physical activities and/or interrupting prolonged sitting with brief bouts of light-intensity physical activity and to reallocate sedentary time and/or light-intensity physical activity toward gradually increasing amounts of purposeful moderate- to vigorous intensity physical activity. |  |
| **HOW WELL**  Planned: If intervention adherence or fidelity was assessed, describe how and by whom, and if any strategies were used to maintain or improve fidelity, describe them. | N/R |  |
| Actual: If intervention adherence or fidelity was assessed, describe the extent to which the intervention was delivered as planned. | N/R |  |
| **Components of Sherifali Diabetes Coaching Model** (2017). | Self-management education and support  Behaviour modification |  |

| Cummings, 2019 | | |
| --- | --- | --- |
| **TIDieR Tool Item** | **Main Paper** | **Other Paper(s)** |
| **BRIEF NAME**  Provide the name or a phrase that describes the intervention. | COMRADE Tailored Cognitive Behavioral Intervention in Type 2 Diabetes |  |
| **WHY**  Describe any rationale, theory, or goal of the elements essential to the intervention. | To evaluate the pragmatic effectiveness of delivering a carefully designed program of severity-tailored CBT and lifestyle counseling on behavioral and glycemic outcomes in patients with T2D and comorbid depressive and/or distress symptoms at screening in a busy rural primary care practice setting.  This behavioral health intervention had the overarching goal of improving the patient’s glycemic control (i.e., HbA1c) as well as improving depressive and/or distress symptoms. |  |
| **WHAT**  Materials: Describe any physical or informational materials used in the intervention, including those provided to participants or used in intervention delivery or in training of intervention providers.  Provide information on where the materials can be accessed (e.g., online appendix, URL). | Consenting patients in both study arms were provided with pictorially rich educational materials about T2D (Living with Diabetes, American College of Physicians, product 11033420E), a weight scale, and a patient friendly Tracking for Success Calendar  developed for daily monitoring of self management behaviors (including fasting blood glucose, weight, medication taken, abbreviated food intake, step count, and mood). |  |
| Procedures: Describe each of the procedures, activities, and/or processes used in the intervention, including any enabling or support activities. | Integrated care delivery model in primary care, bringing together active behavioral intervention with diabetes medical care in the same setting. In the first 6 months of behavioral treatment, all patients in the intervention arm received one individual orientation session and 12 individually tailored behavioral treatment sessions that included the content described below, delivered by the study staff. Session duration ranged from 30 to 60 min depending on treatment content and individual patient needs; patients were not charged for the behavioral intervention. Sessions could also be provided via the telephone. It should be noted that <7% of the intervention arm received one or more phone-based sessions and that these phone-based sessions were provided when barriers prevented patients from attending in-person sessions (e.g., for patients experiencing health or financial difficulties that limited travel). The small-changes lifestyle treatment subgroup included intervention arm patients with low levels of diabetes-related distress and/or depressive symptoms, and the nurse care manager delivered a twice-monthly telephonic intervention focused on lifestyle modifications to improve diabetes and mood based on the small-changes health behavior change model that we have previously described in detail. The CBT subgroup intervention focused on the reduction of depressive and/or RRD symptoms through modification of negative thoughts and problematic behaviors as well as improvement of diabetes self-management strategies. Sessions were delivered by a clinical health psychologist as well as a doctoral student in clinical health psychology. CBT intervention components were guided by two evidence-based treatment manuals for behavioral activation. Session content used cognitive techniques to identify and challenge general and diabetes-specific cognitive distortions that result in maladaptive behavior, in combination with behavioral techniques, including behavioral activation and specific behavior change strategies related to diabetes and/or mood (self-monitoring, sleep hygiene, eating habits, etc.). These sessions occurred face to face (in the primary care clinic) or via telephonic visits with the health psychologist. Patients who met criteria for the CBT subgroup intervention and yet had more intermediate concerns were provided PST. The PST intervention was based on PST for primary care, which is an adapted version of PST specifically for use in primary care clinics. PST, a variant of CBT focuses on the facilitation of effective coping and adaptive problem-solving skills and has been shown to be an effective intervention strategy for improvement in diabetes-specific outcomes. All intervention patients had access to a trained CHW who had extensive experience promoting healthy behaviors for chronic disease management in the targeted region. This CHW provided quarterly telephonic peer support and served as a navigator to community resources that helped patients address logistical challenges to implementing healthy behaviors, problem solving, and accessing healthy food/activity in the target community. |  |
| **WHO PROVIDED**  For each category of intervention provider (e.g., psychologist, nursing assistant), describe their expertise, background and any specific training given. | Trained behavioral providers working together, including a nurse care manager who provided small changes lifestyle coaching, a psychologist and clinical health psychology doctoral student who provided CBT sessions including elements of problem-solving therapy (PST) where indicated, and a community health worker (CHW) who provided navigation and social support. | The primary intervention was delivered by a team of behavioral providers working together including a nurse care manager who provided small-changes lifestyle coaching, a doctoral student in clinical psychology or psychologist who provided cognitive behavioral treatment sessions including elements of problem-solving therapy where indicated, and a community health worker who provided navigation and social support. The nurse care manager was an African American registered nurse from the local area with > 10 years' experience in care management for patients with diabetes. A local African American adult female community health worker with > 10 years' experience in chronic disease care, was hired to provide social support and navigation in the community to acquire needed diabetes resources. All staff hired had strong interpersonal skills including a demonstrated ability to establish rapport quickly with a wide range of people. The nurse manager and other intervention staff received extensive (≥80 h) training by the investigators in all aspects of the intervention including diabetes-specific evaluation and management, depression and distress specific evaluation and management, cognitive behavioral/problem solving therapy intervention components, disease and severity stratification, response evaluation and stepped-care transitions, psychological and medical treatment intensification, working with providers and community partners, and project-specific evaluation. One of the investigators provided regular supervision of the care manager from a behavioral intervention perspective. |
| **HOW**  Describe the modes of delivery (e.g., face-to-face or by some other mechanism, such as internet or telephone) of the intervention and whether it was provided individually or in a group. | Face to face, individually |  |
| **WHERE**  Describe the type(s) of location(s) where the intervention occurred, including any necessary infrastructure or relevant features. | Academic family medicine practice in the southeastern U.S. that provides primary care to a large rural population |  |
| **WHEN and HOW MUCH**  Describe the number of times the intervention was delivered and over what period of time including the number of sessions, their schedule, and their duration, intensity or dose. | One individual orientation session and 12 behavioural treatment sessions, 30-60 minutes, for 12 months |  |
| **TAILORING**  If an intervention was planned to be personalised, titrated or adapted, then describe what, why, when, and how. | The intervention was designed to be tailored or severity stratified such that intervention arm patients were assigned to one of two levels of  behavioral treatment based on the level  of their baseline distress (DDS-17) and  depression (PHQ-9) scores. |  |
| **MODIFICATION**  If an intervention was modified during the course of the study, describe the changes (what, why, when, and how). | N/R |  |
| **HOW WELL**  Planned: If intervention adherence or fidelity was assessed, describe how and by whom, and if any strategies were used to maintain or improve fidelity, describe them. | N/R |  |
| Actual: If intervention adherence or fidelity was assessed, describe the extent to which the intervention was delivered as planned. | N/R |  |
| **Components of Sherifali Diabetes Coaching Model** (2017). | Self-management education and support  Behaviour modification  Psychosocial support |  |

| Jutterström et al, 2016 | | |
| --- | --- | --- |
| **TIDieR Tool Item** | **Main Paper** | **Other Paper** |
| **BRIEF NAME**  Provide the name or a phrase that describes the intervention. | Diabetes intervention in Västerbotten,  part 2 (DIVA-2) with patient-centered self-management support |  |
| **WHY**  Describe any rationale, theory, or goal of the elements essential to the intervention. | The aim of this study was to evaluate the effect of a nurse led patient-centered self-management support in T2D with regard to metabolic changes. The theoretical underpinning of the intervention was the theory of Hernandez about integration and studies on personal understanding in T2D as well as Illness integration and turning points in T2D. |  |
| **WHAT**  Materials: Describe any physical or informational materials used in the intervention, including those provided to participants or used in intervention delivery or in training of intervention providers.  Provide information on where the materials can be accessed (e.g., online appendix, URL). | N/R |  |
| Procedures: Describe each of the procedures, activities, and/or processes used in the intervention, including any enabling or support activities. | In the patient intervention, participants in the GI and II groups were invited to six sessions of 45–90 min each, over a period of up to six months. In the GI groups, the patients reflected aspects of living with type 2 diabetes together and the DSNs acted as a moderator, while she acted as the reflective part in the II groups. The intervention consisted of either discussions in groups or patients or individual conversations with the DSN, depending on the arm of allocation. During the six sessions, the participants were free to discuss issues they considered important in relation to their experiences with the disease. Each session also had a theme; their views of the image of the illness; the meaning of the diagnosis; the illness integration over time; time and the space for management of the illness in daily life; views on the responsibilities of self-management; and lastly, their future prospects for living life with an illness such as T2D. Medical facts were conveyed only when requested by the participants. The II participants met the local diabetes nurse one on-one, while the GI participants met in groups where the diabetes nurse acted as a moderator who made sure that everyone in the group participated in the discussion. |  |
| **WHO PROVIDED**  For each category of intervention provider (e.g., psychologist, nursing assistant), describe their expertise, background and any specific training given. | Diabetes Specialist Nurses (DSNs). Eight DSNs had a postgraduate education in primary health care nursing, one in medicine and surgery; and one lacked a postgraduate education. Nine of the nurses had a university education in diabetes care while the tenth had completed diabetes courses offered by pharmaceutical companies. Before the patient intervention started, ten DSNs from the 9 HCCs participated in a preparatory workshop of approximately 20h that emphasized the patients’ understanding of illness. The patient intervention aimed to improve illness integration and self-management including turning points. The DSNs received a theoretical and practical preparation in patient entered support, illness/disease perspectives and motivating patient-centered communication aimed at supporting illness integration and how to strengthen patients’ self-efficacy for self management. The different perspectives of illness for patients and nurses were reflected upon as an important aspect to be aware of in communication about self-management. DSNs participated in role-playing where one of the DSNs led a session and the other DSNs acted as patients. Afterwards, ambivalence as well as possibilities for self-management were discussed, where the  nurses had opportunities to reflect upon patients’ personal understandings of illness and its relevance to self-management. |  |
| **HOW**  Describe the modes of delivery (e.g., face-to-face or by some other mechanism, such as internet or telephone) of the intervention and whether it was provided individually or in a group. | Group and individual, face-to-face |  |
| **WHERE**  Describe the type(s) of location(s) where the intervention occurred, including any necessary infrastructure or relevant features. | Community-based primary healthcare centers |  |
| **WHEN and HOW MUCH**  Describe the number of times the intervention was delivered and over what period of time including the number of sessions, their schedule, and their duration, intensity or dose. | Six sessions of 45–90 min each, over a period of up to six months. Together with the DSNs, the participants decided when and how often the six sessions should take place, and the intervention period thereby varied between 2 and 6 months. |  |
| **TAILORING**  If an intervention was planned to be personalised, titrated or adapted, then describe what, why, when, and how. | Planned to be personalised: "Nurses had opportunities to reflect upon patients’ personal understandings of illness and its relevance to self-management." When discussing the theme of each session, the authors note “Medical facts were conveyed only when requested by the participants.” |  |
| **MODIFICATION**  If an intervention was modified during the course of the study, describe the changes (what, why, when, and how). | N/R |  |
| **HOW WELL**  Planned: If intervention adherence or fidelity was assessed, describe how and by whom, and if any strategies were used to maintain or improve fidelity, describe them. | N/R |  |
| Actual: If intervention adherence or fidelity was assessed, describe the extent to which the intervention was delivered as planned. | N/R |  |
| **Components of Sherifali Diabetes Coaching Model** (2017). | Self-management education and support  Behaviour modification  Psychosocial support |  |

| Karhula, 2015 | | |
| --- | --- | --- |
| **TIDieR Tool Item** | **Main Paper** | **Other Paper(s)** |
| **BRIEF NAME**  Provide the name or a phrase that describes the intervention. | Telemonitoring and Mobile Phone-Based Health Coaching |  |
| **WHY**  Describe any rationale, theory, or goal of the elements essential to the intervention. | The purpose of this study was to assess the benefits of a structured mobile phone-based health coaching program, supported by a remote monitoring system among chronically ill patients.  The approach followed Wagner’s Chronic Care  Model —one of the key foundational constructs for the approach of chronic care management—and has been developed and tested earlier. | The behavior change component integrated behavior change techniques from the Self-Regulation Theory and supported by evidence (i.e., self-monitoring, goal setting, action planning, and feedback) |
| **WHAT**  Materials: Describe any physical or informational materials used in the intervention, including those provided to participants or used in intervention delivery or in training of intervention providers.  Provide information on where the materials can be accessed (e.g., online appendix, URL). | A self-management guide was given to the patients with the intention to increase their knowledge of their chronic disease.  Each patient in the intervention group received a remote monitoring toolbox to be used in the trial. The toolbox consisted of a mobile phone with specific software, a mobile personal health record (PHR) app, and a set of measurement devices connected to the patient’s PHR account. The mobile PHR app was needed for manual and/or automatic reporting. All patients received a blood pressure meter, which was connectable to the mobile phone via Bluetooth. The mobile PHR app was needed for manual and/or automatic reporting. |  |
| Procedures: Describe each of the procedures, activities, and/or processes used in the intervention, including any enabling or support activities. | Each patient in the intervention group was assigned a personal health coach who called them at regular intervals—every 4 to 6 weeks. A comprehensive evaluation of the patient’s clinical, mental, and social condition was made during the first coaching call and small, achievable health behavior changes were agreed upon with the patient. A self management plan was created based on the targeted changes. During the mobile phone calls that were planned to last for approximately 30 minutes, the health coach provided information, assistance, and support to the patients. The health coaching approach was provided by Pfizer Oy. Each patient in the intervention group received a remote monitoring toolbox to be used in the trial. The mobile PHR app was needed for manual and/or automatic reporting. The health coaches and patients were able to see the patients’ measurements in the PHR and were advised to utilize them during health coaching phone calls. | Patients in the intervention group were called monthly, altogether 10–11 times. After a brief engagement call, there was one broader needs assessment call, followed by monthly coaching calls and finally an evaluation call. In between the coaching calls there was an opportunity for brief follow-up calls, but these were rarely used. The coaching call topics were based on 8 key recommendations of the program, with variations due to individual patient’s preferences.  Call length was also monitored. Calls were found to be long, typically up to 60 min, and they were based on a coach driven information provision model. |
| **WHO PROVIDED**  For each category of intervention provider (e.g., psychologist, nursing assistant), describe their expertise, background and any specific training given. | Health coaches and a health coach supervisor were recruited among the personnel of Eksote. Six coaches were recruited out of 13 applicants. Four of the recruits were working in outpatient care and two in a hospital. The selected coaches continued in their regular positions and worked as health coaches 1 day a week. The health coaches were trained to obtain the needed knowledge about Pfizer’s health coaching model, behavioral management skills, remote monitoring system, and trial procedures. The health coaching model was a solution-oriented  working model where all patients received coaching based on their individual needs. | Seven experienced certified nurses or public health nurses. They were trained for four weeks in a tele-coaching model initially developed by Pfizer Health Solutions (PHS) but modified for the Finnish health care system. Coaches were further trained in Motivational Interviewing techniques of active listening, and using open questions, reflection and summaries, and they all received two individual supervision sessions in self-monitoring and developing their coaching practices. |
| **HOW**  Describe the modes of delivery (e.g., face-to-face or by some other mechanism, such as internet or telephone) of the intervention and whether it was provided individually or in a group. | Telephone, individual |  |
| **WHERE**  Describe the type(s) of location(s) where the intervention occurred, including any necessary infrastructure or relevant features. | N/R |  |
| **WHEN and HOW MUCH**  Describe the number of times the intervention was delivered and over what period of time including the number of sessions, their schedule, and their duration, intensity or dose. |  | 10-11 calls, approx. 30 min to 1 hour in duration 12 months |
| **TAILORING**  If an intervention was planned to be personalised, titrated or adapted, then describe what, why, when, and how. | Planned to be personalised - target changes |  |
| **MODIFICATION**  If an intervention was modified during the course of the study, describe the changes (what, why, when, and how). |  | With these measures, quality (defined as use of structure and Motivational Interviewing techniques, and concrete actions as outcomes of the calls) was improved while call length decreased to approximately 30 min. |
| **HOW WELL**  Planned: If intervention adherence or fidelity was assessed, describe how and by whom, and if any strategies were used to maintain or improve fidelity, describe them. | For quality control and educational purposes, each health coach recorded some of the coaching calls, which were evaluated together with a behavioral science professional once in every 3 months. The equal quality of all health coaches was assured by continuous education and regular meetings, which all the health coaches and the trainer attended. | After the first two months, quality assurance measures were taken in the form of listening to randomly selected 2–3 calls from each coach. |
| Actual: If intervention adherence or fidelity was assessed, describe the extent to which the intervention was delivered as planned. | N/R |  |
| **Components of Sherifali Diabetes Coaching Model** (2017). | Self-management education and support  Behaviour modification |  |

| Naik et al, 2019 | | |
| --- | --- | --- |
| **TIDieR Tool Item** | **Main Paper** | **Other Paper(s)** |
| **BRIEF NAME**  Provide the name or a phrase that describes the intervention. | Telephone-Delivered Collaborative Goal Setting and Behavioral Activation: HOPE intervention |  |
| **WHY**  Describe any rationale, theory, or goal of the elements essential to the intervention. | This study used a learning health care systems approach to screen for and improve  treatment of a high-risk subpopulation. The HOPE approach provides a structured, telephone delivered, collaborative goal-setting intervention for 6 months to enhance behavioral activation targeting depression symptoms and diabetes self-care. The objective of this study was to evaluate  the effectiveness of HOPE for clinically significant improvements in depression and glycemic control compared with enhanced usual care (EUC)—usual diabetes and depression care enhanced by a systems approach to screening for high-risk status. |  |
| **WHAT**  Materials: Describe any physical or informational materials used in the intervention, including those provided to participants or used in intervention delivery or in training of intervention providers.  Provide information on where the materials can be accessed (e.g., online appendix, URL). | Workbooks |  |
| Procedures: Describe each of the procedures, activities, and/or processes used in the intervention, including any enabling or support activities. | Randomized to a 6-month blended diabetes and depression behavioral health coaching program, followed by a 6-month maintenance period without coaching (intervention). Patients and coaches used workbooks that guided telephone conversations and allowed patients to define and track their progress. Primary care physicians received notifications of their patients’ participation, HbA1c results, and PHQ-9 questionnaire outcomes via secure electronic messaging; however, they received no formal training related to the HOPE intervention components. During the first 2 patient sessions, HOPE coaches focused on building rapport, introducing and clarifying values, collaboratively setting initial goals, identifying potential skill sets to address goals, and empowering patients to advocate for their health through active communication with their clinicians. For sessions 3 through 6, participants focused on discrete skill modules (increasing pleasant activities, using thoughts to improve wellness, diet, physical activity, medication management, and relaxation) customized to meet their diabetes and depression goals. Sessions 7 through 9 focused on maintenance skills (reviewing action plans and overcoming barriers). Skills emphasized in the modules were designed to improve diabetes- and depression-related outcomes simultaneously. The HOPE modules stressed the importance of the coach-patient relationship as critical to improvement in participant physical and/or emotional self-management. During months 7 to 12, participants received usual primary care without contact from HOPE coaches. | The coaching model for the HOPE Program was based on the 5As Model for coping with chronic illness. Guided by the 5As (i.e., Assess, Advise, Agree, Assist, Arrange), intervention coaches assisted participants in selecting and refining physical and emotional health goals and in developing action plans to meet their goals. |
| **WHO PROVIDED**  For each category of intervention provider (e.g., psychologist, nursing assistant), describe their expertise, background and any specific training given. | Training of clinicians to deliver a structured telehealth intervention that can be counted as a routine clinical encounter. Twenty-four trained health professionals or coaches (18 female) included psychologists (n = 16), nurses (n = 5), pharmacists (n = 2), and social workers (n = 1). Most (n = 18) were at the MEDVAMC; 6 were at a Veterans Health Administration community-based clinic. Coaches were assigned primarily by similar site of care and availability. Clinician training consisted of facilitated training (two 120-minute teleconference sessions) and subsequent support (mentoring and feedback related to fidelity to the intervention) from a behavioral health expert (N.E.H.), plus monthly peer mentoring with other coaches. |  |
| **HOW**  Describe the modes of delivery (e.g., face-to-face or by some other mechanism, such as internet or telephone) of the intervention and whether it was provided individually or in a group. | All procedures (screening, enrollment,  protocol delivery, and follow-up assessments) were conducted by telephone to ease treatment burden. Participants from both study arms visited the study site only to obtain HbA1c measurements and their usual medical care. |  |
| **WHERE**  Describe the type(s) of location(s) where the intervention occurred, including any necessary infrastructure or relevant features. | Conducted at the MEDVAMC and 6 affiliated community-based outpatient clinics  across Southeast Texas |  |
| **WHEN and HOW MUCH**  Describe the number of times the intervention was delivered and over what period of time including the number of sessions, their schedule, and their duration, intensity or dose. | During the active intervention, the HOPE group received 9 coaching sessions with a trained health professional: biweekly (for 30-40 minutes) from months 1 to 3 and monthly (for 15 minutes) from months 4 to 6.  Individual, telephone |  |
| **TAILORING**  If an intervention was planned to be personalised, titrated or adapted, then describe what, why, when, and how. | Planned to be personalised |  |
| **MODIFICATION**  If an intervention was modified during the course of the study, describe the changes (what, why, when, and how). | N/R |  |
| **HOW WELL**  Planned: If intervention adherence or fidelity was assessed, describe how and by whom, and if any strategies were used to maintain or improve fidelity, describe them. | N/R |  |
| Actual: If intervention adherence or fidelity was assessed, describe the extent to which the intervention was delivered as planned. | N/R |  |
| **Components of Sherifali Diabetes Coaching Model** (2017). | Self-management education and support  Behaviour modification  Psychosocial support |  |

| Odnoletkova, 2016 | | |
| --- | --- | --- |
| **TIDieR Tool Item** | **Main Paper** | **Other Paper(s)** |
| **BRIEF NAME**  Provide the name or a phrase that describes the intervention. | COACH programme |  |
| **WHY**  Describe any rationale, theory, or goal of the elements essential to the intervention. | The aim of the present study was to investigate the effect of the COACH programme on HbA1c and other modifiable diabetes risk factors in people with Type 2 diabetes in a primary care setting in Belgium compared with usual care. The underlying ‘COACH model’ is a continuous quality improvement cycle, which includes bridging the knowledge gap, assertiveness training, setting an action plan and (re)assessment. |  |
| **WHAT**  Materials: Describe any physical or informational materials used in the intervention, including those provided to participants or used in intervention delivery or in training of intervention providers.  Provide information on where the materials can be accessed (e.g., online appendix, URL). | The intervention group received a welcome package containing a nutrition guide, waist circumference metre, BMI calculator and a set for self-monitoring of blood glucose. All study participants received a DVD with educational material on Type 2 diabetes. The laboratory results of the blood analysis were mailed to all study participants and their GPs. |  |
| Procedures: Describe each of the procedures, activities, and/or processes used in the intervention, including any enabling or support activities. | The programme consisted of five telephone sessions of a mean (range) duration of 30 (10–45) min, delivered at a mean (range) interval of 5 (3–8) weeks by a certified diabetes nurse educator (hereafter referred to as the ‘coach’) after a 5-day training course. It consisted of an update of the best practice guidelines for the management of Type 2 diabetes, motivational interviewing techniques and software program use. The coaches analysed patient risk profiles based on the baseline assessment data and consulted GPs on the individual therapeutic goals before the start of the programme. After each session, a written coaching report was prepared by the coach and sent to the participant and his/her GP. The reports contained a comparison between the recommended and the actual outcomes for diabetes risk factors and an agreed action plan to bridge any resulting gap. |  |
| **WHO PROVIDED**  For each category of intervention provider (e.g., psychologist, nursing assistant), describe their expertise, background and any specific training given. | Certified diabetes nurse educator (hereafter referred to as the ‘coach’) after a 5-day training course | Prior to the intervention, they undergo 5-days training in up-to date clinical guidelines on diabetes self-management and how to give patients the motivation and the skill to improve their risk factors. Coaches are also trained in the use of the COACH Program software for patient administration. |
| **HOW**  Describe the modes of delivery (e.g., face-to-face or by some other mechanism, such as internet or telephone) of the intervention and whether it was provided individually or in a group. | Telephone, individual |  |
| **WHERE**  Describe the type(s) of location(s) where the intervention occurred, including any necessary infrastructure or relevant features. | Home  Primary care settings |  |
| **WHEN and HOW MUCH**  Describe the number of times the intervention was delivered and over what period of time including the number of sessions, their schedule, and their duration, intensity or dose. | Five telephone sessions of a mean (range) duration of 30 (10–45) min, delivered at a  mean (range) interval of 5 (3–8) weeks – approximately 25 weeks (range 15 to 40 weeks). |  |
| **TAILORING**  If an intervention was planned to be personalised, titrated or adapted, then describe what, why, when, and how. | Planned to be personalised | Coaching is focused on eliminating the knowledge and treatment gap and motivating the patient to apply the appropriate lifestyle and medical therapy. Each session is used as the foundation for the next contact. The coaching model is a continuous five-stage coaching cycle. |
| **MODIFICATION**  If an intervention was modified during the course of the study, describe the changes (what, why, when, and how). | N/R |  |
| **HOW WELL**  Planned: If intervention adherence or fidelity was assessed, describe how and by whom, and if any strategies were used to maintain or improve fidelity, describe them. | The intervention quality control measurements included a review of coaching reports by one of the present authors (I.O.) during the first 3 months and selectively thereafter, audio-recording of several sessions, and weekly programme  monitoring briefings. |  |
| Actual: If intervention adherence or fidelity was assessed, describe the extent to which the intervention was delivered as planned. |  | Of 252 patients available for a follow-up analysis, 97.5 % reported being satisfied. Interviews were held with 16 patients, 17 general practitioners (GPs) and all nurses involved (n = 6). |
| **Components of Sherifali Diabetes Coaching Model** (2017). | Self-management education and support  Behaviour modification |  |

| Sherifali et al, 2021 | | |
| --- | --- | --- |
| **TIDieR Tool Item** | **Main Paper** | **Other Paper(s)** |
| **BRIEF NAME**  Provide the name or a phrase that describes the intervention. | Diabetes Health Coaching on Glycemic Control and Quality of Life in Adults Living with Type 2 Diabetes | Telephone-based diabetes health coaching |
| **WHY**  Describe any rationale, theory, or goal of the elements essential to the intervention. | The purpose of this study was to evaluate the effect of a 12-month intensive telephone support health coaching intervention on change in glycated hemoglobin (A1C) levels over time, as well as QoL in adults living with T2DM in the community setting | The goal of this study was to evaluate the effects of an evidence-informed diabetes health-coaching intervention via telephone. The outcomes considered included: A1C, BMI, QoL (generic and diabetes), self-care activities, adherence to the intervention and cost-effectiveness. |
| **WHAT**  Materials: Describe any physical or informational materials used in the intervention, including those provided to participants or used in intervention delivery or in training of intervention providers.  Provide information on where the materials can be accessed (e.g., online appendix, URL). | All participants in the study received access to usual diabetes education (individual or group) provided by nurses and/or dietitians, typically every 3 to 6 months, along with community resources and a study provided accelerometer. | Participants were provided with community-based resources to support diabetes self-care activities such as physical-activity programming. Participants were also provided with personal journals to record ideas, goals and interactions with the coaches. Finally, participants were provided with an outline of coaching sessions (1/week for 6 months and then 1/month for another 6 months). Participants were required to have access to or to have a telephone (land line or cellular). |
| Procedures: Describe each of the procedures, activities, and/or processes used in the intervention, including any enabling or support activities. | The intervention group received diabetes health coaching, delivered by a registered nurse/certified diabetes educator, with training in the DHC model, motivational interviewing and behavioural design, specifically a certification in Tiny Habits coaching, which emphasizes small, positive habits customized to one’s environment, ability and motivation. Weekly phone calls by the coach occurred in the first 6 months and monthly phone calls in the last 6 months. The topic or agenda of each telephone call was determined by the participant or as agreed upon from the previous coaching session. The full intervention description for replication has been reported previously. | Participants were encouraged to connect with their coaches via telephone and voice-mail messages. If agreed-upon times for telephone interactions were missed, the diabetes health coach left up to 3 messages/phone calls to connect. No further attempts were made. |
| **WHO PROVIDED**  For each category of intervention provider (e.g., psychologist, nursing assistant), describe their expertise, background and any specific training given. | Registered nurse/certified diabetes educator, with training in the DHC model, motivational interviewing and behavioural design, specifically a certification in Tiny Habits coaching, which emphasizes small, positive habits customized to one’s environment, ability, and motivation. | The diabetes health coach is a graduation-prepared nurse and a certified diabetes educator trained in the Diabetes Health Coach model, behaviour design and motivational interviewing. |
| **HOW**  Describe the modes of delivery (e.g., face-to-face or by some other mechanism, such as internet or telephone) of the intervention and whether it was provided individually or in a group. | Individual phone calls | The intervention was provided exclusively via telephone to individuals. |
| **WHERE**  Describe the type(s) of location(s) where the intervention occurred, including any necessary infrastructure or relevant features. |  | All coach and participant interactions occurred remotely via telephone. |
| **WHEN and HOW MUCH**  Describe the number of times the intervention was delivered and over what period of time including the number of sessions, their schedule, and their duration, intensity or dose. | Weekly calls for first 6 months, monthly calls for next 6 months duration | The intervention was 1 year in duration, with 1/week telephone interactions for the first 6 months, followed by 1/month in the last 6 months. Each telephone interaction was aimed to be about 15 minutes in length. |
| **TAILORING**  If an intervention was planned to be personalised, titrated or adapted, then describe what, why, when, and how. |  | Frequency was tailored to participants’ needs as well as the duration (e.g., 5 to 10 min). The topic of discussion for each telephone call was determined by the participant, specifically: 1) case management and monitoring; 2) self-management education; 3) psychosocial support; and 4) behaviour modification. |
| **MODIFICATION**  If an intervention was modified during the course of the study, describe the changes (what, why, when, and how). | N/R |  |
| **HOW WELL**  Planned: If intervention adherence or fidelity was assessed, describe how and by whom, and if any strategies were used to maintain or improve fidelity, describe them. |  | Operational fidelity, as demonstrated by adherence to the coaching intervention, was defined as the proportion of participants who utilized the coaching as outlined (100% attendance to all sessions). Theoretic fidelity to the Diabetes Health Coach model was assessed through discussions and observations by the research coordinator and principal investigator. |
| Actual: If intervention adherence or fidelity was assessed, describe the extent to which the intervention was delivered as planned. | N/R |  |
| **Components of Sherifali Diabetes Coaching Model** (2017). | Self-management education and support  Behaviour modification  Psychosocial support  Case management and monitoring |  |

| Varney, 2014 | | |
| --- | --- | --- |
| **TIDieR Tool Item** | **Main Paper** | **Other Paper(s)** |
| **BRIEF NAME**  Provide the name or a phrase that describes the intervention. | Hospital-based telephone coaching |  |
| **WHY**  Describe any rationale, theory, or goal of the elements essential to the intervention. | Comparing with usual care, the effects of a 6-month telephone coaching intervention on glycaemic control, risk factor status and adherence to self-care and monitoring requirements, both at the intervention’s conclusion (6 months), and in the post-intervention period (12 months). |  |
| **WHAT**  Materials: Describe any physical or informational materials used in the intervention, including those provided to participants or used in intervention delivery or in training of intervention providers.  Provide information on where the materials can be accessed (e.g., online appendix, URL). | N/R |  |
| Procedures: Describe each of the procedures, activities, and/or processes used in the intervention, including any enabling or support activities. | 6 months of telephone coaching, delivered by a dietitian with experience in cardiovascular disease and T2DM (JV). Initial coaching sessions occurred within 2 weeks of randomisation and subsequent sessions occurred approximately monthly. Coach session duration was flexible, determined by time required to establish participant goals. Typically, initial and follow-up sessions took 45 and 20 min respectively. Intervention group participants received 6.0 (95% CI 5.5–6.5, range 4–9) coaching sessions. Advice given in coaching sessions was consistent with Australian guidelines. During initial coaching sessions, a diet history was taken. Participants were encouraged to follow a low saturated fat, high fibre diet, with 50% of energy from carbohydrates, and were encouraged to exercise for 150 min per week. Risk factor status and adherence to monitoring requirements were based on information collected at baseline. For treatment goals and risk factors not at target levels, the dietary, lifestyle, and medication changes required to improve these parameters were discussed. The coach delivering the intervention did not prescribe medication, therefore, participants were advised to discuss medication changes with their general practitioner (GP). Discrepancies between participants’ adherence to self-care activities  (Diet and physical activity) and monitoring requirements (foot checks, eye checks and vaccinations) were highlighted, and the appropriate management schedule was explained. Participant goals were then agreed, be this a change in diet or a podiatry appointment for an overdue foot examination. Following each coaching session, the participant and their GP received a letter summarizing the participant’s goals. During subsequent coaching sessions, progress towards treatment goals, risk factor status, adherence to self-care and monitoring requirements were reassessed. If goals were not achieved, barriers to goal attainment were identified, an action plan addressing these barriers was agreed and new goals were established. This process was repeated throughout the intervention. |  |
| **WHO PROVIDED**  For each category of intervention provider (e.g., psychologist, nursing assistant), describe their expertise, background and any specific training given. | Dietitian with experience in cardiovascular disease and T2DM (area of specialty) |  |
| **HOW**  Describe the modes of delivery (e.g., face-to-face or by some other mechanism, such as internet or telephone) of the intervention and whether it was provided individually or in a group. | Telephone coaching, individual |  |
| **WHERE**  Describe the type(s) of location(s) where the intervention occurred, including any necessary infrastructure or relevant features. | Home  Diabetes Clinic of St Vincent’s Hospital Melbourne (STV), an Australian public teaching hospital |  |
| **WHEN and HOW MUCH**  Describe the number of times the intervention was delivered and over what period of time including the number of sessions, their schedule, and their duration, intensity or dose. | 6 coaching session (range 4-9), Flexible timing (typically, initial and follow-up sessions took 45 and 20 min) |  |
| **TAILORING**  If an intervention was planned to be personalised, titrated or adapted, then describe what, why, when, and how. | Planned to be personalised |  |
| **MODIFICATION**  If an intervention was modified during the course of the study, describe the changes (what, why, when, and how). | N/R |  |
| **HOW WELL**  Planned: If intervention adherence or fidelity was assessed, describe how and by whom, and if any strategies were used to maintain or improve fidelity, describe them. | Reasons for attrition were not collected |  |
| Actual: If intervention adherence or fidelity was assessed, describe the extent to which the intervention was delivered as planned. | N/R |  |
| **Components of Sherifali Diabetes Coaching Model** (2017). | Self-management education and support  Psychosocial support  Case management and monitoring |  |

| Young et al, 2020 | | |
| --- | --- | --- |
| **TIDieR Tool Item** | **Main Paper** | **Other Paper(s)** |
| **BRIEF NAME**  Provide the name or a phrase that describes the intervention. | A nurse coaching program using motivational interviewing paired with mobile health (mHealth) technology on diabetes self-efficacy and self-management for persons with type 2 diabetes. |  |
| **WHY**  Describe any rationale, theory, or goal of the elements essential to the intervention. | This study examined the impact of a novel intervention using MI-based nurse health coaching combined with wearable activity  trackers that integrate patient-generated activity data into the patient’s electronic health record (EHR) to improve health among adults with type 2 diabetes. We hypothesized that individuals randomized to the intervention group would show overall improved self-efficacy compared with individuals in the usual care group. |  |
| **WHAT**  Materials: Describe any physical or informational materials used in the intervention, including those provided to participants or used in intervention delivery or in training of intervention providers.  Provide information on where the materials can be accessed (e.g., online appendix, URL). | Wearable tracking device (initially, Basis Peak, then Garmin VivoSmart Heart Rate [HR]), standard health care visits with providers, and access to classes, resources, services (i.e., diabetes management and weight loss education, electronic learning videos, and care coordination), iPod Touch to participants who did not already possess this technology, access to MyFitnessPal if participant wanted to log and track nutritional consumption |  |
| Procedures: Describe each of the procedures, activities, and/or processes used in the intervention, including any enabling or support activities. | The intervention included (1) nurse health coaching and (2) mHealth technology to track PGHD and integrate these data into the EHR.  We paired each participant with a nurse health coach who delivered 6 individual sessions using a counseling style based on the concepts of MI. Sessions were structured to promote mutual goal setting, enhance self-efficacy in health behavior change, and assist individuals to derive meaning from data to reinforce choices and behaviors. Two RN researchers with nurse coaching experience in diabetes audited 8 of the 158 (5%) of the participant sessions and scored the coach using the MITI. They provided timely feedback to the coaches during weekly debriefing sessions, reviewed scores, and discussed optimization strategies by reviewing scenarios. The participants had an in-person orientation with the nurse coach, followed by telephone sessions every 2 weeks for 3 months (6 contacts total). The initial MI session elicited motivations and set goals with tracking metrics to gauge the progress toward goals at subsequent sessions. Throughout the sessions, the coaches encouraged the participants to identify facilitators and barriers to achieving their health goals. |  |
| **WHO PROVIDED**  For each category of intervention provider (e.g., psychologist, nursing assistant), describe their expertise, background and any specific training given. | The nurse health coaches for the intervention were 3 registered nurses (RNs) with experience in both health coaching and management of chronic disease. To promote fidelity to the intervention and a common approach to coaching participants, the nurses received core training in MI-based coaching and diabetes management. All the RN health coaches delivering the intervention completed the HealthSciences Institute’s Registered Health Coach (RHC) and Chronic Care Professional training programs (www.healthsciences.org). A final performance evaluation using the Motivational Interviewing Treatment Integrity (MITI) 3.1.1 global scale evaluation tool confirmed health coaching competency before the receipt of the RHC certificate. Nurses also completed a refresher course in diabetes management through the American Association of Diabetes Educators as well as the standard health system orientation on policies, procedures, and EHR training. |  |
| **HOW**  Describe the modes of delivery (e.g., face-to-face or by some other mechanism, such as internet or telephone) of the intervention and whether it was provided individually or in a group. | Individual, telephone |  |
| **WHERE**  Describe the type(s) of location(s) where the intervention occurred, including any necessary infrastructure or relevant features. | The participants had an in-person orientation with the nurse coach, followed by telephone sessions. We provided in-person or telephonic technical support to all participants—including the usual care group participants—throughout the duration of the study. |  |
| **WHEN and HOW MUCH**  Describe the number of times the intervention was delivered and over what period of time including the number of sessions, their schedule, and their duration, intensity or dose. | 6 individual sessions over 3 months/12 weeks  Length/duration not reported |  |
| **TAILORING**  If an intervention was planned to be personalised, titrated or adapted, then describe what, why, when, and how. | Planned to be personalised |  |
| **MODIFICATION**  If an intervention was modified during the course of the study, describe the changes (what, why, when, and how). | Early in the intervention period, we experienced an unexpected recall of the Basis Peak activity tracking device because of a safety issue that required identifying and selecting a replacement device. The study team, in collaboration with the advisory boards, worked diligently and promptly to identify, test, and select a replacement (Garmin VivoSmart HR) and then distribute the new device to the participants in the intervention arm of the study, providing technical support to these participants as needed. This recall affected 79 participants; most of these participants received and were oriented to their new devices within 2 weeks of the recall. |  |
| **HOW WELL**  Planned: If intervention adherence or fidelity was assessed, describe how and by whom, and if any strategies were used to maintain or improve fidelity, describe them. | Two RN researchers with nurse coaching experience in diabetes audited 8 of the 158 (5%) of the participant sessions |  |
| Actual: If intervention adherence or fidelity was assessed, describe the extent to which the intervention was delivered as planned. | N/R |  |
| **Components of Sherifali Diabetes Coaching Model** (2017). | Self-management education and support  Behaviour modification  Psychosocial support  Case management and monitoring |  |

Legend:

N/R = information about the element is not reported/not sufficiently reported.

Sherifali Diabetes Coaching Model comprises: (i) personal case management and monitoring, emphasizing process of care issues and system navigation related to diabetes; (ii) diabetes self-management education and support, highlighting the need for knowledge, skill acquisition, and problem solving related to day-today management; (iii) behavior modification, goal setting and reinforcement, using motivational interviewing and theories to facilitate goal setting, attainment, and behavior change; and (iv) general psychosocial support, leveraging active listening and empathy to provide support. *Sherifali D. Diabetes coaching for individuals with type 2 diabetes: A state-of-the-science review and rationale for a coaching model. J Diabetes. 2017 Jun;9(6):547-554. doi: 10.1111/1753-0407.12528.*

# GRADE evidence rating: Health coaching interventions compared to usual care for the management of patients with T2DM.

| **Certainty assessment** | | | | | | | **№ of patients** | | **Effect** | **Certainty** | **Importance** |
| --- | --- | --- | --- | --- | --- | --- | --- | --- | --- | --- | --- |
| **№ of studies** | **Study design** | **Risk of bias** | **Inconsistency** | **Indirectness** | **Imprecision** | **Other considerations** | **Health coaching** | **Usual care** | **Absolute (95% CI)** |  |  |

| **Quality of life (Immediate-post treatment)** | | | | | | | | | | | | | | | | | | | | | | |
| --- | --- | --- | --- | --- | --- | --- | --- | --- | --- | --- | --- | --- | --- | --- | --- | --- | --- | --- | --- | --- | --- | --- |
| 4^t^ | randomised trials | | serious^b^ | | not serious^i^ | not serious | | serious^l^ | | none^e^ | | 724 | | 643 | | SMD **0.03 SD higher** (0.08 lower to 0.14 higher) | | ⨁⨁◯◯ Low | | CRITICAL | | |
| **Quality of life (Long-term follow-up) (follow-up: range 6 months to 12 months)** | | | | | | | | | | | | | | | | | | | | | |  |
| 2^u^ | | randomised trials | | serious^b^ | serious^n^ | | not serious | | serious^l^ | | none^e^ | | 369 | | 400 | | SMD **0.07 SD lower** (0.32 lower to 0.19 higher) | | ⨁◯◯◯ Very low | | CRITICAL |  |
| **Depression / Distress (Immediate-post treatment)** | | | | | | | | | | | | | | | | | | | | | |  |
| 5^v^ | | randomised trials | | serious^b^ | not serious^w^ | | not serious | | not serious^d^ | | none^e^ | | 579 | | 584 | | SMD **0.21 SD lower** (0.41 lower to 0.02 lower) | | ⨁⨁⨁◯ Moderate | | CRITICAL |  |
| **Depression / Distress (Long-term follow-up) (follow-up: range 6 months to 12 months)** | | | | | | | | | | | | | | | | | | | | | |  |
| 4^x^ | | randomised trials | | serious^b^ | serious^n^ | | not serious | | serious^l^ | | none^e^ | | 485 | | 484 | | SMD **0.11 SD lower** (0.38 lower to 0.16 higher) | | ⨁◯◯◯ Very low | | CRITICAL |  |
| **HbA1C (Immediate-post treatment)** | | | | | | | | | | | | | | | | | | | | | |  |
| 7^a^ | | randomised trials | | serious^b^ | not serious^c^ | | not serious | | not serious^d^ | | none^e^ | | 957 | | 841 | | SMD **0.24 SD lower** (0.38 lower to 0.09 lower) | | ⨁⨁⨁◯ Moderate | | CRITICAL |  |
| **HbA1C (Long-term follow-up) (follow-up: range 6 months to 12 months)** | | | | | | | | | | | | | | | | | | | | | |  |
| 4^f^ | | randomised trials | | serious^b^ | not serious^c^ | | not serious | | serious^g^ | | none^e^ | | 457 | | 382 | | SMD **0.22 SD lower** (0.46 lower to 0.01 higher) | | ⨁⨁◯◯ Low | | CRITICAL |  |
| **Body mass index (Immediate-post treatment)** | | | | | | | | | | | | | | | | | | | | | |  |
| 2^o^ | | randomised trials | | serious^b^ | not serious^i^ | | not serious | | not serious^q^ | | none^e^ | | 294 | | 302 | | SMD **0.19 SD lower** (0.35 lower to 0.03 lower) | | ⨁⨁⨁◯ Moderate | | CRITICAL |  |
| **Body mass index (Long-term follow-up) (follow-up: range 6 months to 12 months)** | | | | | | | | | | | | | | | | | | | | | |  |
| 3^j^ | | randomised trials | | serious^b^ | not serious^i^ | | not serious | | serious^l^ | | none^e^ | | 365 | | 314 | | SMD **0.04 SD lower** (0.19 lower to 0.11 higher) | | ⨁⨁◯◯ Low | | CRITICAL |  |
| **Waist Circumference (Immediate-post treatment)** | | | | | | | | | | | | | | | | | | | | | |  |
| 3^r^ | | randomised trials | | serious^b^ | not serious^i^ | | not serious | | not serious^q^ | | none^e^ | | 331 | | 250 | | SMD **0.24 SD lower** (0.41 lower to 0.07 lower) | | ⨁⨁⨁◯ Moderate | | CRITICAL |  |
| **Waist Circumference (Long-term follow-up) (follow-up: mean 6 months)** | | | | | | | | | | | | | | | | | | | | | |  |
| 2^s^ | | randomised trials | | serious^b^ | not serious^i^ | | not serious | | very serious^p^ | | none^e^ | | 127 | | 68 | | SMD **0.10 SD lower** (0.36 lower to 0.17 higher) | | ⨁◯◯◯ Very low | | CRITICAL |  |
| **Body Weight (Immediate-post treatment)** | | | | | | | | | | | | | | | | | | | | | |  |
| 5^h^ | | randomised trials | | serious^b^ | not serious^i^ | | not serious | | not serious^d^ | | none^e^ | | 664 | | 584 | | SMD **0.19 SD lower** (0.3 lower to 0.08 lower) | | ⨁⨁⨁◯ Moderate | | CRITICAL |  |
| **Body Weight (Long-term follow-up) (follow-up: range 6 months to 12 months)** | | | | | | | | | | | | | | | | | | | | | |  |
| 2^o^ | | randomised trials | | serious^b^ | not serious^i^ | | not serious | | serious^p^ | | none^e^ | | 273 | | 282 | | SMD **0.06 SD lower** (0.23 lower to 0.1 higher) | | ⨁⨁◯◯ Low | | CRITICAL |  |
| \| **Certainty assessment** \| \| \| \| \| \| \| **№ of patients** \| \| **Effect** \| **Certainty** \| **Importance** \| \| --- \| --- \| --- \| --- \| --- \| --- \| --- \| --- \| --- \| --- \| --- \| --- \| \| **№ of studies** \| **Study design** \| **Risk of bias** \| **Inconsistency** \| **Indirectness** \| **Imprecision** \| **Other considerations** \| **Health coaching** \| **Usual care** \| **Absolute (95% CI)** \|   **Systolic blood pressure (Immediate-post treatment)** | | | | | | | | | | | | | | | | | | | | | |  |
| 5^h^ | | randomised trials | | serious^b^ | not serious^i^ | | not serious | | not serious^d^ | | none^e^ | | 659 | | 585 | | SMD **0.28 SD lower** (0.4 lower to 0.16 lower) | | ⨁⨁⨁◯ Moderate | | CRITICAL |  |
| **Systolic blood pressure (Long-term follow-up) (follow-up: range 6 months to 12 months)** | | | | | | | | | | | | | | | | | | | | | |  |
| 3^j^ | | randomised trials | | serious^b^ | not serious^i^ | | not serious | | not serious^d^ | | none^e^ | | 368 | | 315 | | SMD **0.38 SD lower** (0.53 lower to 0.23 lower) | | ⨁⨁⨁◯ Moderate | | CRITICAL |  |
| **Diastolic blood pressure (Immediate-post treatment)** | | | | | | | | | | | | | | | | | | | | | |  |
| 4^k^ | | randomised trials | | serious^b^ | not serious^c^ | | not serious | | serious^l^ | | none^e^ | | 592 | | 513 | | SMD **0.18 SD lower** (0.4 lower to 0.04 higher) | | ⨁⨁◯◯ Low | | CRITICAL |  |
| **Diastolic blood pressure (Long-term follow-up) (follow-up: range 6 months to 12 months)** | | | | | | | | | | | | | | | | | | | | | |  |
| 3^j^ | | randomised trials | | serious^b^ | not serious^i^ | | not serious | | serious^l^ | | none^e^ | | 368 | | 315 | | SMD **0.01 SD lower** (0.16 lower to 0.14 higher) | | ⨁⨁◯◯ Low | | CRITICAL |  |
| **Total Cholesterol (Immediate-post treatment)** | | | | | | | | | | | | | | | | | | | | | |  |
| 4^m^ | | randomised trials | | serious^b^ | not serious^c^ | | not serious | | serious^l^ | | none^e^ | | 593 | | 512 | | SMD **0.12 SD lower** (0.31 lower to 0.07 higher) | | ⨁⨁◯◯ Low | | CRITICAL |  |
| **Total Cholesterol (Long-term follow-up) (follow-up: range 6 months to 12 months)** | | | | | | | | | | | | | | | | | | | | | |  |
| 3^j^ | | randomised trials | | serious^b^ | not serious^c^ | | not serious | | serious^l^ | | none^e^ | | 368 | | 314 | | SMD **0.10 SD higher** (0.19 lower to 0.39 higher) | | ⨁⨁◯◯ Low | | CRITICAL |  |
| **Low density lipoprotein (Immediate-post treatment)** | | | | | | | | | | | | | | | | | | | | | |  |
| 4^k^ | | randomised trials | | serious^b^ | not serious^i^ | | not serious | | serious^l^ | | none^e^ | | 591 | | 507 | | SMD **0.11 SD lower** (0.23 lower to 0.02 higher) | | ⨁⨁◯◯ Low | | CRITICAL |  |
| **Low density lipoprotein (Long-term follow-up) (follow-up: range 6 months to 12 months)** | | | | | | | | | | | | | | | | | | | | | |  |
| 3^j^ | | randomised trials | | serious^b^ | not serious^c^ | | not serious | | serious^l^ | | none^e^ | | 364 | | 307 | | SMD **0.07 SD higher** (0.14 lower to 0.27 higher) | | ⨁⨁◯◯ Low | | CRITICAL |  |
| **High density lipoprotein (Immediate-post treatment)** | | | | | | | | | | | | | | | | | | | | | |  |
| 4^k^ | | randomised trials | | serious^b^ | not serious^i^ | | not serious | | serious^l^ | | none^e^ | | 594 | | 511 | | SMD **0.06 SD lower** (0.18 lower to 0.06 higher) | | ⨁⨁◯◯ Low | | CRITICAL |  |
| **High density lipoprotein (Long-term follow-up) (follow-up: range 6 months to 12 months)** | | | | | | | | | | | | | | | | | | | | | |  |
| 3^j^ | | randomised trials | | serious^b^ | not serious^i^ | | not serious | | serious^l^ | | none^e^ | | 364 | | 307 | | SMD **0.08 SD lower** (0.23 lower to 0.07 higher) | | ⨁⨁◯◯ Low | | CRITICAL |  |
| **Triglycerides (Immediate-post treatment)** | | | | | | | | | | | | | | | | | | | | | |  |
| 4^k^ | | randomised trials | | serious^b^ | serious^n^ | | not serious | | serious^l^ | | none^e^ | | 594 | | 499 | | SMD **0.30 SD lower** (0.73 lower to 0.13 higher) | | ⨁◯◯◯ Very low | | CRITICAL |  |
| **Triglycerides (Long-term follow-up) (follow-up: range 6 months to 12 months)** | | | | | | | | | | | | | | | | | | | | | |  |
| 3^j^ | | randomised trials | | serious^b^ | not serious^i^ | | not serious | | serious^l^ | | none^e^ | | 368 | | 346 | | SMD **0.09 SD lower** (0.24 lower to 0.05 higher) | | ⨁⨁◯◯ Low | | CRITICAL |  |

**CI:** confidence interval; **SMD:** standardised mean difference

#### Explanations

a. Sherifali, 2021; Balducci, 2019; Cummings, 2019; Naik, 2019; Odnoletkova, 2016; Karhula, 2015; Varney, 2014.

b. Studies were rated as unclear risk of bias with lack of reporting or concerns regarding blinding of participants and outcome assessment, and incomplete outcome reporting.

c. The confidence intervals overlap with moderate statistical heterogeneity observed across studies.

d. The sample size is adequate (≥300) in each arm. The effect estimate is precise with narrow confidence intervals.

e. Too few studies (n<10) to assess publication bias.

f. Naik, 2019; Jutterstrom, 2016; Odnoletkova, 2016; Varney, 2014.

g. The sample size is not adequate (<300) in each arm, and the effect estimate is imprecise with confidence intervals including the no effect.

h. Balducci, 2019; Cummings, 2019; Odnoletkova, 2016; Karhula, 2015; Varney, 2014.

i. The confidence intervals overlap with minimal statistical heterogeneity observed across studies.

j. Odnoletkova, 2016; Jutterstrom, 2016; Varney, 2014.

k. Balducci, 2019; Odnoletkova, 2016; Karhula, 2015; Varney, 2014.

l. The sample size is adequate (≥300) in each arm. The summary 95% confidence intervals include the no effect value of "0", however, they do not cross an effect size of 0.5 in either direction.

m. Balducci, 2019; Odnoletkova, 2016; Karhula, 2015; Varney, 2014.

n. The confidence intervals don't overlap, and the direction of effect is not consistent with substantial heterogeneity observed across studies.

o. Odnoletkova, 2016; Varney, 2014.

p. The sample size is inadequate (<300) in each arm, and the effect estimate is imprecise with confidence intervals including the no effect.

q. The sample size is inadequate (<300) in each arm, however, the effect estimate is precise with narrow confidence intervals.

r. Balducci, 2019; Karhula, 2015; Varney, 2014.

s. Jutterstrom, 2016; Varney, 2014.

t. Sherifali, 2021; Young, 2020; Odnoletkova, 2016; Karhula, 2015.

u. Young, 2020; Odnoletkova, 2016.

v. Young, 2020; Cummings, 2019; Naik, 2019; Odnoletkova, 2016; Varney, 2014.

w. High statistical heterogeneity, however, the direction of effect is consistent across most studies and confidence intervals overlap.

x. Young, 2020; Naik, 2019; Odnoletkova, 2016; Varney, 2014.

# Meta-analysis for Non-Significant Outcomes


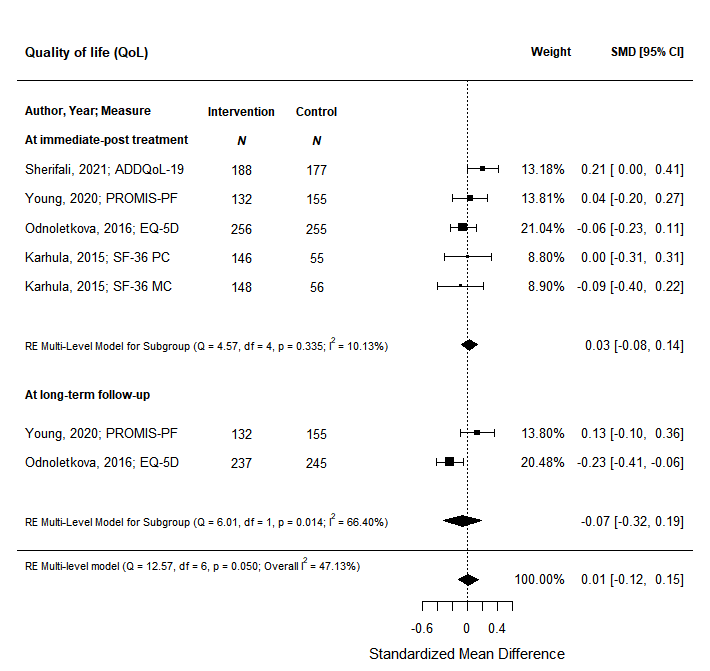


Figure 1. The effect of coaching interventions on quality of life outcomes


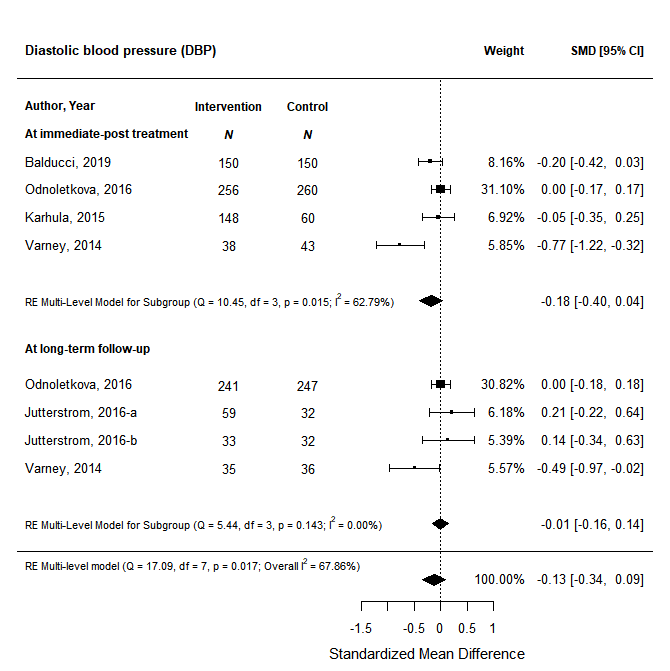


Figure 2. The effect of coaching interventions on diastolic blood pressure


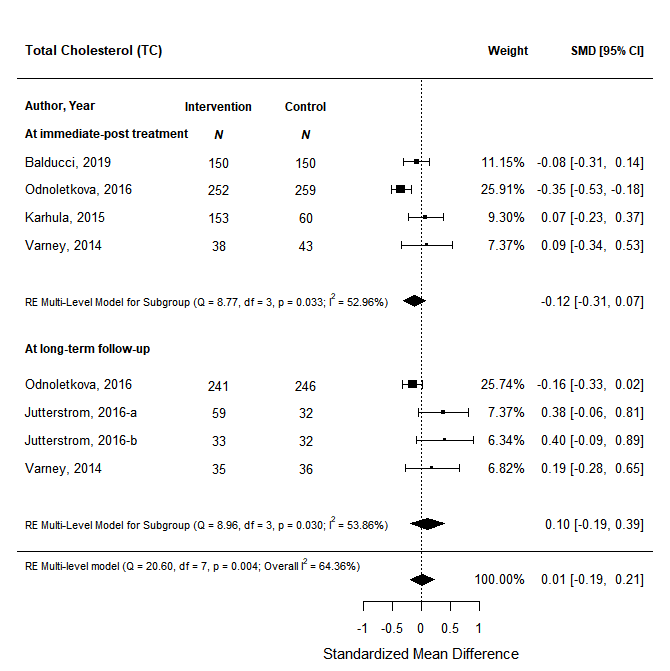


Figure 3. The effect of coaching interventions on total cholesterol


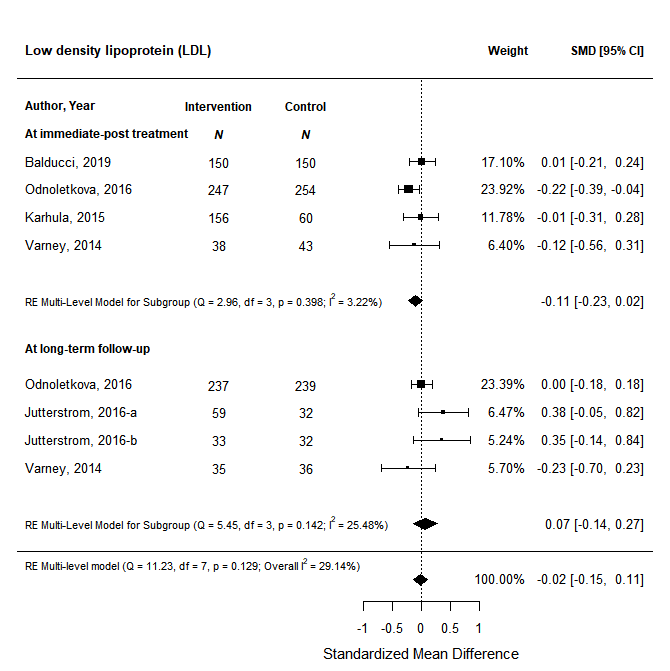


Figure 4. The effect of coaching interventions on LDL


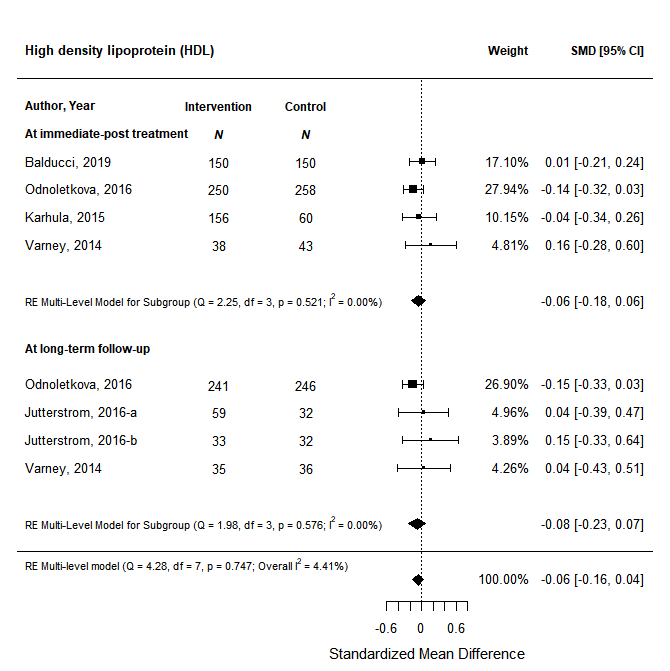


Figure 5. The effect of coaching interventions on HDL


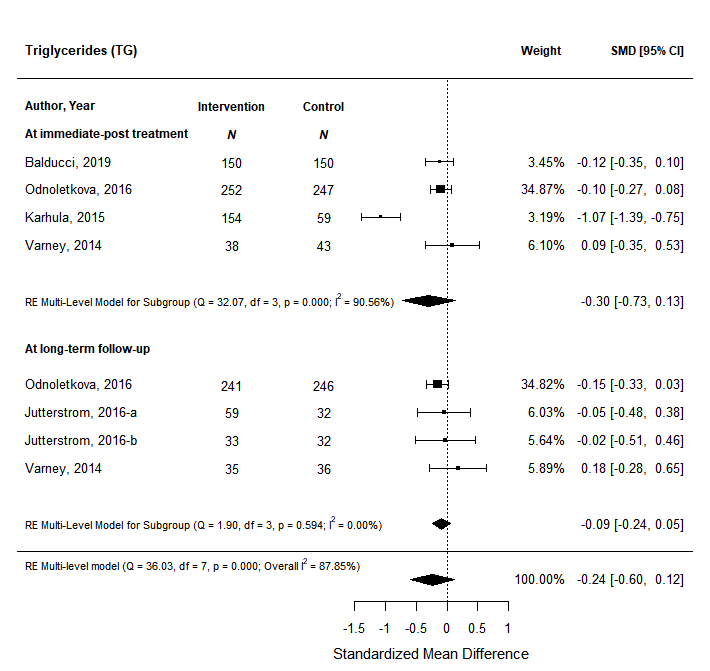


Figure 6. The effect of coaching interventions on triglycerides
